# One-Pot Synthesis of Novel Pyrimidine Derivatives with Potential Antidiabetic Activity Through Dual α-Glucosidase and α-Amylase Inhibitors

**DOI:** 10.3390/molecules30132857

**Published:** 2025-07-04

**Authors:** Ohood Al-Shehri, Samar Abubshait, Muhammad Nawaz, Mohamed S. Gomaa, Haya A. Abubshait

**Affiliations:** 1 Department of Chemistry, College of Science, Imam Abdulrahman Bin Faisal University, P.O. Box 1982, Dammam 31441, Saudi Arabia; 2 Basic and Applied Scientific Research Center, Imam Abdulrahman Bin Faisal University, P.O. Box 1982, Dammam 31441, Saudi Arabia; 3 Department of Nano-Medicine Research, Institute for Research and Medical Consultations (IRMC), Imam Abdulrahman Bin Faisal University, P.O. Box 1982, Dammam 31441, Saudi Arabia; mnnmuhammad@iau.edu.sa; 4 Department of Pharmaceutical Chemistry, College of Clinical Pharmacy, Imam Abdulrahman Bin Faisal University, P.O. Box 1982, Dammam 31441, Saudi Arabia; msmmansour@iau.edu.sa; 5 Basic Sciences Department, Deanship of Preparatory Year and Supporting Studies, Imam Abdulrahman Bin Faisal University, P.O. Box 1982, Dammam 31441, Saudi Arabia; habubshait@iau.edu.sa

**Keywords:** pyrimidine derivatives, phenyl isothiocyanate, anti-diabetes activity, α-glucosidase, α-amylase

## Abstract

This study describes the synthesis of heterocyclic derivatives containing multiple nitrogen atoms serving as important moieties for developing novel antidiabetics through a simple synthetic pathway. We herein describe the synthesis and characterization of novel pyrimidine derivatives using one-pot reactions in a catalyst-free and efficient manner through a two-stage process involving the synthesis of 2-amino-4-hydrazinyl-6-methoxy pyrimidine, followed by a reaction with phenyl isothiocyanate derivatives. The structures of all the new compounds were confirmed via physical and spectral analysis. Furthermore, we evaluated the synthesized pyrimidine derivatives’ biological activities in relation to their potential roles as novel anti-diabetic agents by testing their activity profiles against the enzymes α-glucosidase and α-amylase. Compound **4** expressed the highest level of activity against α-glucosidase and α-amylase, with a greater inhibitory concentration (IC_50_ of 12.16 ± 0.12 µM and IC_50_ 11.13 ± 0.12 µM) compared to that of acarbose (IC_50_ = 10.60 ± 0.17 µM and IC_50_ = 11.30 ± 0.12 µM), which is widely used as a standard antidiabetic drug. The primary structure activity relationship analysis identified the impact of an electron- withdrawing group, especially with respect to fluorine on inhibitory activity. This was further confirmed in molecular docking studies, which demonstrated that both compounds exhibited similar inhibition patterns and emphasized the significance of incorporating a lipophilic electron-withdrawing substituent on the phenyl ring, along with the 2,4-diaminopyrimidine scaffold.

## 1. Introduction

Because of their vast range of applications, heterocyclic compounds have been the subject of a great deal of research in the last ten years. Pyridazine, pyrimidine, and pyrazine are examples of heterocyclic compounds that have a six-membered ring with two nitrogen atoms, known as diazines. These derivatives play a significant role in biological, pharmacological, industrial, and medical processes [1]. The derivatives of pyrimidine, which are especially important in biological systems, include nucleic acids [2]. The pyrimidine moiety is a crucial building block found in many different pharmaceutical types. Since it is an essential component of DNA and RNA, pyrimidine has been extracted from hydrolyzed nucleic acids and offers a variety of pharmacological characteristics [3,4,5]. Although pyrimidine derivatives have been described as having broad biological potential, most published research reports concentrate on pharmacological properties, such as their antimicrobial [6], antifungal [7,8], antivirus [9,10], anticancer [11,12,13], anti-HIV [14,15], antimutator [16,17], anti-inflammatory, anti-leishmanial, anti-malarial [2], antioxidant [18], anticholinergic [19], anti-diabetic [20], and α-glucosidase-inhibitory activity [21], and use as a component of anti-GD2-immunoliposomes for the therapeutic treatment of neuroblastoma [22]. Against this backdrop is diabetes mellitus, a chronic and non-infectious metabolic disease characterized by hyperglycemia, i.e., a high blood glucose level. The prevalence of diabetes mellitus is alarming; about 415 million people worldwide are estimated to have diabetes [23], and this number is expected to increase to about 642 million by 2040 [24,25]. Enzymes such as α-amylase and α-glucosidase hydrolyze carbohydrates into glucose in the blood. α-glucosidase inhibitors thus reduce the absorption of glucose from the small intestine by inhibiting the activity of α-glucosidase secreted by the intestinal epithelium and salivary glands to hydrolyze oligosaccharides into simple sugar. The inhibition of α-amylase and α-glucosidase delays glucose absorption, thereby lowering postprandial blood glucose levels, constituting an effective therapeutic approach to the treatment of diabetes. This capacity is particularly important since there is always a great demand for the design and development of novel, safe, and effective anti-diabetic compounds [20,26,27,28]. Pyrimidine derivatives represent an attractive scaffold with which to develop new and effective lead compounds with potential anti-diabetic activity. Nucleophilic substitutions of chloropyrimidine with amides, amines, alcoholates, sulfides, and Grignard reagents are good applications for the preparation of functionalized pyrimidines [29]. This work provides a detailed approach to the synthesis and characterization of novel pyrimidine derivatives using a one-pot reaction, in a catalyst-free and efficient manner, for the synthesis of 2-amino-4-hydrazinyl-6-methoxy pyrimidine and its subsequent reaction with phenyl isothiocyanate derivatives. However, the sustainability aspect of our synthetic approach, which involves a one-pot, catalyst-free method, aligns with green chemistry principles by reducing reaction steps, eliminating the need for metal catalysts, and minimizing waste generation—making it both environmentally and economically favorable for the synthesis of bioactive molecules [30]. Physical and spectral analyses were used to characterize the synthesized compounds. Furthermore, the biological activity and pharmacological profiles of the pyrimidine derivatives were determined by assessing their activity against the enzymes α-glucosidase and α-amylase. Using this investigational approach, we aimed to develop lead compounds with dual inhibitory effects against both α-amylase and α-glucosidase that would aid in the treatment of diabetes [26,31,32,33]. We describe in this study the synthesis, characterization, potential antidiabetic activity through dual α-glucosidase and α-amylase inhibition, and molecular docking investigations of a new series of potential inhibitors bearing a 2,4-diaminopyrimidine scaffold.

## 2. Results and Discussion

### 2.1. Chemistry

**Scheme 1:**
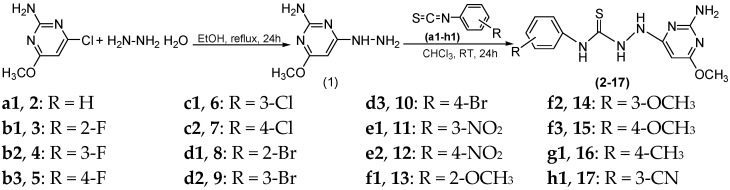
Preparation method of 2-amino-6-methoxypyrimidine derivatives (**2**–**17**).

Pyrimidine derivatives have been reported to have a wide range of biological actions with anti-microbial [34,35,36], anti-oxidant and anti-cancer [37,38], anti-infective [39], anti-neuroinflammatory [40], anti-Alzheimer [41], anti-diabetic [42,43], anti-tubercular [44], bioactive [45,46], and medicinal approaches [47,48]. Preparation of 2-amino-4-hydrazinyl-6-methoxy pyrimidine (**1**) was carried out by refluxed 2-amino-4-chloro-6-methoxypyrimidine and hydrazine hydrate in the presence of ethanol as a solvent. The reaction mixture was refluxed at 80–85 °C for 24 h and then left for 24 h at 25 °C. Precipitates were filtered off and then washed with cold ethanol to give compounds (**1**). The second step involved the reaction of 2-amino-4-hydrazinyl-6-methoxy pyrimidine (**1**) with phenyl isothiocyanate derivatives at room temperature (Scheme 1).

The assigned structures for synthesized compound (**1**) are recognized by FTIR, ^1^H NMR,^13^C NMR, and mass spectroscopic techniques. While the FTIR spectra showed bands in the region between 2952, 2862 cm^−1^ for (C-H) stretching, a band in the area (3030, 3060) cm^−1^ for aromatic (=C-H), and (C=C) stretching bands appeared, respectively. In addition, a new peak appeared for (NH-NH_2_) at regions (3427, 3139) cm^−1^, and a band of the (-Cl) at 785–540 group disappeared. Furthermore, ^1^H NMR spectra have a singlet band δ3.36 related to (-OC**H**_3_) and 5.88 for (-C**H**) pyrimidine, while bands of -N**H**-, N**H**_2_ appeared at δ 4.25, 4.80, and 10.22 ppm. Moreover, the ^13^C NMR spectra showed a major carbon band at δ 71.3 corresponding to the (-O**C**H_3_) group and bands in the region δ93.4–171.1 for pyrimidine carbon. The reaction of hydrazinylpyrimidine (**1**) with phenyl isothiocyanate derivatives (**a1**–**h1**) in chloroform at room temperature for 24 h gave 2-amino-6-methoxypyrimidine derivatives (**2**–**17**)**,** respectively. The observed selectivity of the hydrazinyl group (–NH–NH_2_) over the amino group (–C–NH_2_) in the reaction of 2-amino-4-hydrazinyl-6-methoxy pyrimidine (**1**) with phenyl isothiocyanate derivatives can be attributed to both electronic and steric factors. The terminal –NH_2_ group of the hydrazine moiety is more nucleophilic than the aromatic amino group due to its higher electron density and reduced resonance delocalization with the pyrimidine ring. This makes the hydrazinyl –NH_2_ more reactive toward electrophiles such as isothiocyanates. Additionally, the hydrazinyl group is positioned more freely in space compared to the ring-bound amino group, reducing steric hindrance during nucleophilic attack. As a result, under the applied reaction conditions, the isothiocyanate preferentially reacts with the hydrazinyl –NH_2_ group, selectively forming the corresponding thiosemicarbazide intermediate. This behavior is consistent with previously reported chemoselectivity patterns in hydrazine-containing heterocycles [49,50,51,52]. The FTIR spectra of thiourea derivatives (**2**–**17**) showed new (-NH-(C=S)-NH) stretching bands. However, compounds showed new bands related to new groups. Furthermore, in the ^1^H, ^13^C NMR spectra of (**2**–**17**)**,** major bands -OCH_3_ existed for pyrimidine aromatic protons.

### 2.2. Biological Evaluation

#### Antidiabetic Activity

The biological evaluation of the synthesized pyrimidine derivatives against α-glucosidase and α-amylase enzymes is summarized in Table 1. The variations in inhibitory activity among the compounds are likely attributed to the presence of different functional groups. Compound **17** exhibited low inhibitory activity, with IC_50_ values of 42.19 ± 0.13 µM and 40.16 ± 0.11 µM against both α-glucosidase and α-amylase, compared to the standard drug acarbose (IC_50_: 09.33 ± 0.04 µM and 0.15 ± 0.06 µM). In contrast, compound **4** showed remarkable inhibitory potential against both enzymes (IC_50_: 12.16 ± 0.12 µM and 11.13 ± 0.12 µM), as did compound **6**, with IC_50_ values of 16.13 ± 0.11 µM and 15.17 ± 0.07 µM. Compound **9** also displayed significant inhibition, recording IC_50_ values of 19.11 ± 0.11 µM and 18.16 ± 0.08 µM against α-glucosidase and α-amylase. In the case of compound **11**, inhibitory potential against *α*-glucosidase and *α*-amylase was as follows: IC_50_ 22.11 ± 0.13 µM and 21.14 ± 0.12 µM, respectively. Similarly, compound **14** showed inhibitory potential against *α*-glucosidase and *α*-amylase with IC_50_ values of 30.12 ± 0.13 µM and 33.18 ± 0.14 µM, respectively. On the other hand, compound **16** exhibited low activity, with IC_50_ values of 36.14 ± 0.12 µM and 34.24 ± 0.16 µM against both enzymes. Among all tested derivatives, compound **4**, featuring a fluoro substituent, displayed the strongest inhibitory effect. This enhanced activity may be attributed to the high electronegativity of the fluorine atom and its favorable interaction within the enzyme active sites.

### 2.3. Computational Simulation Studies

The primary interpretation of the enzyme-binding assay results was that an electron-withdrawing group is superior to an electron-donating substituent on the phenyl ring (compounds **4**, **6**, **9**, and **11** compared to compound **16**), possibly because electron withdrawal increases the hydrogen bonding donor ability of the thiourea nitrogen and helps to increase the quantity of the thiourea tautomer relative to the iminothiol tautomer. Substitution in the meta position yielded active compounds through its inductive effect on the thiourea nitrogen. The nature of the electron withdrawing substituent also contributes to this activity, as groups with better hydrogen acceptor ability were more active compounds (compound **4**, compared to compounds **6**, **9**, and **11**). The hydrogen bonding capacity of the fluorine compared to the chlorine, the bromine, and the nitro could explain the relative difference in affinity between those compounds. A more lipophilic substituent also seems to be more favorable when we compare compounds **4**, **6**, **9,** and **11**, which have F, Cl, Br, and NO_2_ substituents, respectively, with compound **17**, which has the CN substituent. In conclusion, an electron-withdrawing, relatively lipophilic group with hydrogen bonding capability is favored in the meta position. The affinity of the tested compounds to α-glucosidase and α-amylase was also studied using molecular docking to gain insight into the potential binding interactions that could drive further molecular optimization. The accuracy of Glide in predicting the binding positions for α-glucosidase and α-amylase was validated by redocking the crystal ligands and calculating the relative mean standard deviation (RMSD) for the predicted positions. The results showed good accuracy for Glide in predicting binding to α-glucosidase and α-amylase active sites, with RMSDs of 2.8 and 2.4 for α-glucosidase and α-amylase, respectively. The XP score yielded by Glide together with the free energy of binding (MM-GBSA dG bind) were analyzed and correlated with the experimental IC_50_s. XP mode is a more advanced and realistic mode of Glide that can screen out false positives, and it is designed to identify active compounds that bind to a particular conformation of a receptor. Explicit water molecules are docked into protein ligand complexes, and descriptors based on the interactions of these water molecules with the ligands and proteins are used as a measure of whether the complex is physically realistic. Penalties are assigned to inadequately solvated structures. GlideScore XP also specifically calculates hydrophobic interactions such that underestimated hydrophobic effects are offset and up to several kcal/mol of additional binding energy can be conferred in favorable cases. GlideScore XP also includes improvements to the scoring of hydrogen bonds as well as the ability to detect buried polar groups, and π-cation and π-π stacking interactions. The MMG-BSA was used to calculate ligand binding energies and ligand strain energies using the Prime module on Maestro. The ranking of the ligands based on the calculated binding energies (via MM-GBSA dG Bind) can be expected to agree reasonably well with a ranking based on experimental binding affinity, particularly in the case of congeneric series. As the MM-GBSA binding energies are approximate free energies of binding, a more negative value indicates stronger binding. MM-GBSA provides reasonable free energy calculations and yields higher enrichment factors than docking calculations [53]. MM-GBSA is therefore more reliable for prioritizing compounds for experimental testing. The MM-GBSA binding free energies were estimated as the total free energies of the protein ligand complex minus the energies of individual ligands and receptors [54,55]. The energy term refers to the sum of the van der Waals, electrostatic, General Born solvation, and surface area energies [56]. The results showed that the MM-GBSA dG binding scores identified the most active and least active compounds (compound **4** compared to compound **17**) with respect to *α*-glucosidase. However, the XP scores were more correlated with the experimental IC_50_s (compounds **4**, **6**, and **9** compared to compounds **14**, and **17**). In the case of α-amylase, the MM-GBSA dG binding scores were more discriminative (compounds **4**, **6**, and **11** compared to compound **14**) than the XP scores. The relatively narrow range of the experimental activity of the compounds and the XP and MM-GBSA scores could be a reason for the imperfect alignment (Table 2).

**Figure 1:**
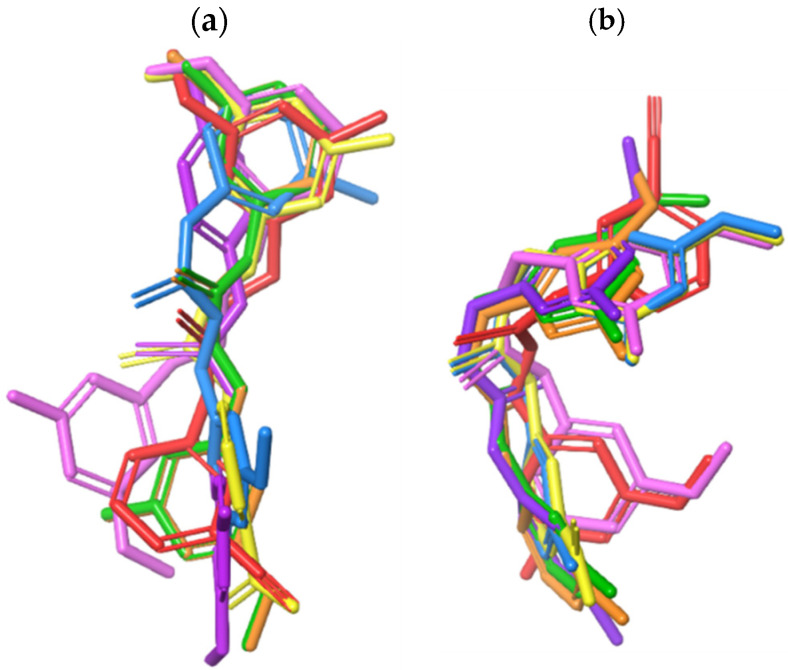
Binding poses of compounds **4** (green), 6 (brown), **9** (blue), **11** (yellow), **14** (pink), **16** (magenta), and **17** (red) in (**a**) α-glucosidase (PDB code: 3W37) and (**b**) α-amylase (PDB code: 4GQR) active sites.

In this case, the binding interaction analysis is of outmost importance for obtaining better insights into the structure activity relationship of the pyrimidine derivatives for *α*-glucosidase and α-amylase inhibition. The binding position of the tested compounds showed good alignment with each other as well as with the crystal ligands and the active sites of α-glucosidase and α-amylase active sites (Figure 1). The calculated relative mean standard deviation (RMSD) for the tested pyrimidine analogues showed excellent alignment of the binding poses for the most active compounds, namely compounds **4**, **6**, **9**, and **11,** with an RMSD below 2 in both active sites (Table 3). The least active compound, **17**, showed the highest deviation in both enzymes. These results suggest that the most active compounds are more capable of engaging in the crucial binding interactions required for the inhibition of both enzymes.

**Figure 2:**
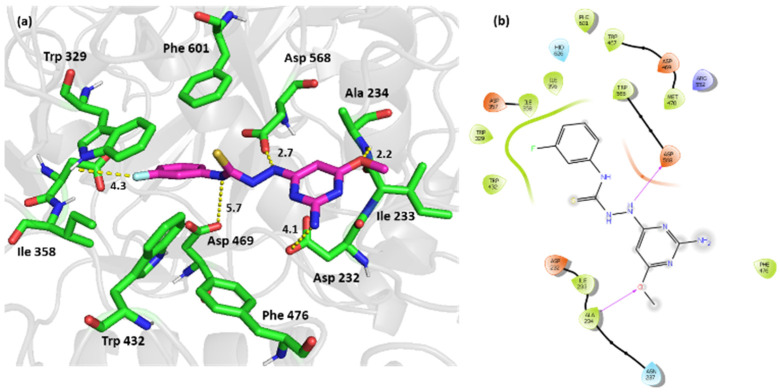
(**a**) Three-dimensional representation of the binding interactions between compound **4** and α-glucosidase (PDB code: 3W37) active site and the interacting residues were shown as cartoon, sticks (carbon atoms colored in magenta), and sticks (carbon atom colored in green), respectively. Potential electrostatic interactions are represented as yellow dotted lines and are measured in Angstrom. (**b**) Two-dimensional ligand–protein binding interactions between compound **4** and α-glucosidase (PDB code: 3W37) active site.

The active site of α-glucosidase (PDB code: 3W37) consists of four subsites (−1, +1, +2, and +3). Subsites −1 and +1 contain two important catalytic residues: Asp 469 and Asp 568. Subsites +2 and +3 contain the following residues: Asp 232, Ile 233, Ala 234, Phe 236, Asn-237, Trp 329, Ile 358, Trp 432, Phe 476, and Phe 601; together, these form a hydrophobic barrier at the entrance of the active site pocket. Trp 329 is a conserved residue that offers catalytic specificity to α-1,6-glucosidic linkages [57]. Compound **4** interacted with the two important catalytic residues, Asp 469, and Asp 568, through hydrogen bonding (Figure 2). The phenyl ring with its lipophilic fluorine substituent was located in the hydrophobic entrance, and potential interactions with π-π staking and halogen bonding with Trp 239 were noted. This finding supports the primary structure activity analysis that postulated that a lipophilic substituent is more favored at this position. It seems that in our pyrimidine derivatives, the phenyl ring and its substituent facilitate the entry of the compounds into the active site and their stability therein and help in acquiring the proper orientation of the di- amino pyrimidine scaffold to interact with the key catalytic residues. Potential hydrogen bonding was also noted between the primary amino group and Asp 232 and the methoxy group and Ala 234.

**Figure 3:**
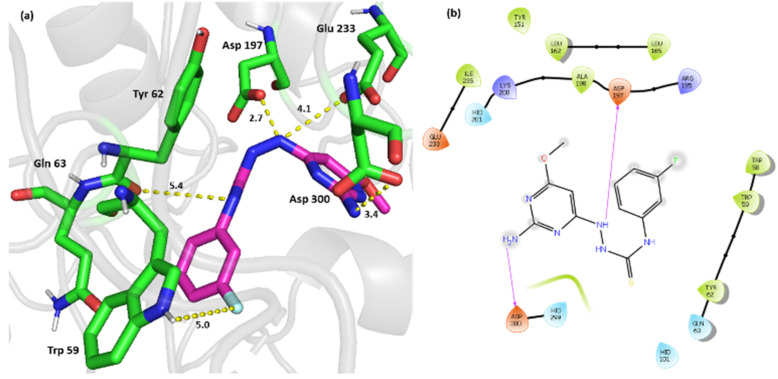
(**a**) Three-dimensional representation of the binding interactions between compound **4** and α-amylase (PDB code: 4GQR) active site and the interacting residues, which are shown as cartoon, sticks (carbon atoms colored in magenta), and sticks (carbon atom colored in green), respectively. Potential electrostatic interactions are represented as yellow dotted lines and are measured in Angstrom. (**b**) Two-dimensional ligand–protein binding interactions between compound **4** and α-amylase (PDB code: 4GQR) active site.

The active site of α-amylase contains two important catalytic residues, Asp 197 and Glu 233. Asp 197 is known to act as the catalytic nucleophile in the hydrolysis reaction catalyzed by α-amylase, while Glu 233 acts as the acid-base catalyst. Asp 300 is another important residue that optimizes the orientation of the substrate in the active site [58]. Compound 4 formed two important hydrogen bonds with the two catalytic residues, Asp 197 and Alu 233. The phenyl ring, as depicted in α-glucosidase, interacted with Trp 59 at the entrance of the active site through π-π staking and halogen bonds. Additional hydrogen bonds were formed with other key residues in the active site, including Asp 300 and Gln 63. In the previous discussion, one can note a close similarity regarding our pyrimidine derivatives’ inhibition mechanisms for both enzymes. This finding is very compatible with the aligned activities of 147 of our pyrimidine derivatives for both enzymes (Figure 3).

**Figure 4:**
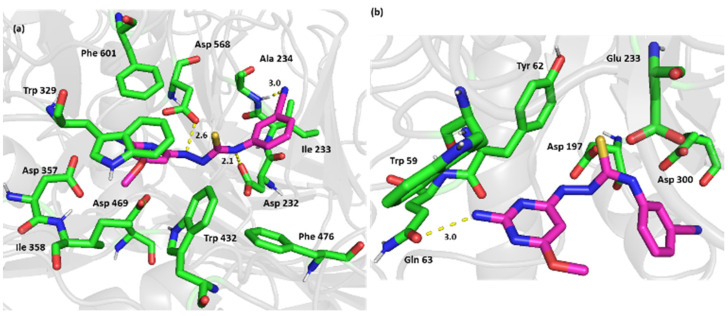
3D binding interactions of compound **17** with (**a**) α-glucosidase (PDB: 3W37) and (**b**) α-amylase (PDB: 4GQR). Interacting residues are shown as cartoon and sticks (C atoms in magenta and green). Electrostatic interactions are indicated by yellow dashed lines (in Å).

Furthermore, the binding of the least active compounds showed different orientations in both enzymes with the phenyl ring and its more hydrophilic cyano substituent, and unlike the fluoro phenyl in compound **4**, it occurred far from the hydrophobic gates. Compound **17** engaged in hydrogen bonding with Asp 232 and Asp 568 in α-glucosidase and with Gln 62 in α-amylase, interactions that are not as crucial as the interactions engaged in in the case of compound **4**. This finding explains the lower activity of compound **17** attributed to its low stability and low-affinity orientation in the active sites of both enzymes (Figure 4).

Potential electrostatic interactions are represented as yellow dotted lines and measured in Angstroms. Three-dimensional representations of the binding interactions between compound **17** and the active site of α-amylase (PDB code: 4GQR) and the interacting residues are shown as digital representations in the form of cartoon, sticks (carbon atoms colored in magenta), and sticks (carbon atom colored in green), respectively. Potential electrostatic interactions are represented as yellow dotted lines and are measured in Angstrom. 

In conclusion, our research has identified 2,4-diaminopyrimidine as a new scaffold for the dual inhibition of α-glucosidase and α-amylase. This scaffold is essential in binding the key catalytic residues at the active sites of α-glucosidase and α-amylase. It should be attached to a lipophilic aromatic system to give it optimal stability and the best orientation at the active sites. The substitution of a lipophilic substituent on the phenyl ring increases its activity. The spacer between the di-amino pyrimidine scaffold and the aromatic ring should contain heteroatoms with good hydrogen bonding ability.

## 3. Materials and Methods

### 3.1. General Methods

All chemicals were supplied from Sigma-Aldrich (St. Louis, MO, USA). Melting points were determined via the Stuart SMP10 Digital Advanced MP apparatus (Cole-Parmer Ltd., Stone, UK) and are uncorrected. FTIR spectra were carried out on PerkinElmer (FTIR spectrometer, PerkinElmer Inc., Waltham, MA, USA), which mixed without using anhydrous KBr. The NMR spectra were recorded by JEOL RESONANCE spectrometer (Akishima, Japan) 500 MHZ by using DMSO-*d*_6_ as the solvent and chemical shifts (δ) and were recorded in parts per million (ppm) using tetramethyl silane (TMS) as the internal standard. The mass spectra were carried out on LC/MS spectroscopy (LCMS-8040 Shimadzu, Kyoto, Japan, model CAT-30A). Serial no. L20574900241 AE, 220–240 v~50/60 HZ 300 VA was used to record LCMS spectral data for samples. Elemental microanalysis was done on a Carlo Erba analyzer model 110 (Carlo Erba Instruments, Milan, Italy). Also used were α-amylase Assay Kit (Colorimetric) ab102523 (Abcam, Cambridge, MA, USA), α-glucosidase Activity Assay Kit (Colorimetric) ab102523 (Abcam, Cambridge, MA, USA), and Elisa: BioTek (Winooski, VT, USA) introduces Synergy Neo2 high performance Multi-Mode Microplate Reader.

### 3.2. General Procedures of Synthesis New Compounds

#### 3.2.1. Synthesis of 2-Amino-4-hydrazinyl-6-methoxypyrimidine (**1**)

An amount of 5.0 g (31.33 mmol) of 2-Amino-4-chloro-6-methoxy-pyrimidine was weighed and transferred into a round-bottomed flask with 150 mL of ethanol and left for stirring, then 50 mL hydrazine hydrate were added to the reaction solution. The reaction mixture was refluxed overnight in oil bath. After reaction completion, the product was transferred into a beaker and left for evaporation. The obtained product was washed with diethyl ether (30 mL 3 times) and dried at room temperature and had the following characteristics: White powder; yield (88.03%); m.p.: 238–239 °C; FT-IR (KBr, ν, cm^−1^): 3427, 3139 (-NHNH_2_), 3398, 3343 (-N-H_st_), 1582 (-N-H_bend_), 1079 (C-N_st Ar_), 3030, 3060 (=C-H st), 2952, 2862 (-C-H_st_), 1265 (-O-CH3st); ^1^H NMR, δ (ppm) in DMSO-*d*_6_: 3.36 (s, 3H, -OC**H**_3_), 4.25 (br, 2H, -N**H**_2_), 4.80 (br, 2H, -N**H**_2 Pyrimidine_), 5.88 (s, 1H, -C**H**_Pyrimidine_), 10.22 (br, 1H, -N**H**-); ^13^C NMR, δ (ppm) in DMSO-*d*_6_: 71.3 (1C, -O**C**H_3_), 93.4 (1C, -C=**C**H-C=N-_Pyrimidine_), 163.3 (1C, =**C**-NH_2 Pyrimidine_), 168.9 (1C, =**C**-OCH_3 Pyrimidine_), 171.1 (1C, =**C**-NH-NH_2 Pyrimidine_); MS (*m*/*z*): 155.0 (M^+^, 100%); Anal. calcd. for C_5_H_9_N_5_O (MW:155.16): C, 38.70; H, 5.85; N, 45.14; found: C, 38.71; H, 5.87; N, 45.15.

#### 3.2.2. Reaction of 2-Amino-4-hydrazinyl-6-methoxypyrimidine with Phenyl Isothiocyanate Derivatives to Give Compounds (**2**–**17**)

An amount of 0.086 g (0.5 mmol) of 2-amino-4-hydrazinyl-6-methoxypyrimidine (**1**) was weighed and transferred into a 25 mL round-bottomed flask along with 10 mL of chloroform. Then, 0.6 mmol of phenyl isothiocyanate, as given in the list (0.081 g) of phenyl isothiocyanate (**a**), (0.092 g) of 2-fluorophenyl isothiocyanate (**b1**), 3-fluorophenyl isothiocyanate (**b2**)**,** 4-fluorophenyl isothiocyanate (**b3**), (0.102 g) of 3-chlorophenyl isothiocyanate (**c1**), 4-chlorophenyl isothiocyanate (**c2**), (0.128 g) of 2-bromophenyl isothiocyanate (**d1**), 3-bromophenyl isothiocyanate (**d2**), 4-bromophenyl isothiocyanate (**d3**), (0.108 g) of 3-nitrophenyl isothiocyanate (**e1**), 4-nitrophenyl isothiocyanate (**e2**), (0.099 g) of 2-methoxyphenyl isothiocyanate (**f1**), 3-methoxyphenyl isothiocyanate (**f2**), 4-methoxyphenyl isothiocyanate (**f3**), (0.096 g) of 3-cyanophenyl isothiocyanate (**g1**), and (0.090 g) of *p*-tolyl isothiocyanate (**h1**)**,** was added to the flask, and the reaction mixture was stirred for 24 h at room temperature. After 24 h, the reaction mixture was transferred into a beaker, and the solvent was evaporated completely. Products were washed with diethyl ether (20–30 mL), dried at room temperature, and stored at 4 °C. The following were produced:

##### 2-(2-Amino-6-methoxypyrimidin-4-yl)-N-phenylhydrazine-carbothioamide (**2**)

White powder; yield (64.29%); m.p.: 188–189 °C; FT-IR (KBr, ν, cm^−1^): 3180, 3300 (N-H st), 1470 (C-N), 726 (C=S), 3498, 3344 (-N-H_st_), 1652 (-N-H_bend_), 1064 (C-N_st_ Ar), 3029, 3060 (=C-H_st_), 2960, 2862 (-C-H_st_), 1231(-OCH3st); ^1^H NMR, δ (ppm) in DMSO-*d*_6_: 3.73 (s, 3H, -OC**H**_3_), 5.39 (br, 2H,-N**H**_2_), 9.48 (br, 2H, =N**H**-N**H**-), 10.14 (br, 1H, -N**H**-CS), 5.80 (s, 1H, =C**H**-_Pyrimidine_), 7.12–7.85 (m, 5H,=C**H-**_Phenyl_); ^13^C NMR, δ (ppm) in DMSO-*d*_6_: 53.2 (1C, -O**C**H_3_), 70.9 (1C, -C=**C**H-C=N-_Pyrimidine_), 125.7, 128.4 (5C, =**C**-, =**C**H-_Phenyl_), 139.8 (1C, =C-NH-_Phenyl_), 160.40 (1C, =**C**-NH_2 Pyrimidine_), 163.2 (1C, =**C**-NH-N=_Pyrimidine_), 166.1 (1C, =**C**-OCH_3_,_Pyrimidine_), 169.4 (1C, -**C**=S); MS (*m*/*z*): 290.00 (M^+^, 100.0%); Anal. calcd. for C_12_H_14_N_6_OS (MW: 290.34); C, 49.64; H, 4.86; N, 28.95; S, 11.04%; found: C, 49.64; H, 4.85; N, 28.98; S, 11.02%.

##### 2-(2-Amino-6-methoxypyrimidin-4-yl)-N-(2-fluorophenyl)-hydrazine Carbothioamide (**3**)

Beige powder; yield (74.60%); m.p.: 200–201 °C; FT-IR (KBr, ν, cm^−1^): 3180, 3300 (N-H_st_), 1428 (C-N), 783 (C=S), 3498, 3344 (-N-H_st_), 1582 (-N-H_bend_); 1074 (C-N_stAr_), 3029, 3060 (=C-H_st_), 2960, 2862 (-C-H_st_), 1264(-OCH3st), 1100 (C-F); ^1^H NMR, δ (ppm) in DMSO-*d*_6_: 3.74 (s, 3H, -OC**H**_3_), 6.03 (s, 1H, =C**H**-_Pyrimidine_), 7.19–7.68 (m, 4H, =C**H**-_Phenyl_), 4.74 (br, 2H, -N**H**_2_), 8.85 (br, 2H, =N**H**-N**H**-), 9.48 (br, 1H, -N**H**-CS); ^13^C NMR, δ (ppm) in DMSO-*d*_6_: 53.3 (1C, -O**C**H_3_), 70.5 (1C, -C=**C**H-_Pyrimidine_), 114.0, 124.2, 127.8, 129.8 (5C, =**C**-, =**C**H-_Phenyl_), 156.1 (1C, =**C**-F _Phenyl_), 159.4 (1C, =**C**-NH_2 Pyrimidine_), 163.1 (1C, =**C**-NH-N=_Pyrimidine_), 165.8 (1C, =**C**-OCH_3 Pyrimidine_), 182.2 (1C, -**C**=S); MS (*m*/*z*): 308.0 (M^+^, 100.0%); Anal. calcd. for C_12_H_13_FN_6_OS (MW: 308.33); C, 46.74; H, 4.25; N, 27.26; S, 10.40; F, 6.16%; found: C, 46.77; H, 4.24; N, 27.27; S, 10.41; F, 6.17%.

##### 2-(2-Amino-6-methoxypyrimidin-4-yl)-N-(3-fluorophenyl)hydrazine Carbothioamide (**4**)

Beige powder; yield (57.38%); m.p.: 184–185 °C; FT-IR (KBr, ν, cm^−1^): 3170, 3301(N-H_st_), 1430 (C-N), 784 (C=S), 3488, 3341 (-N-H_st_), 1588 (-N-H_bend_), 1070 (C-N_stAr_), 3026, 3066 (=C-H_st_), 2960, 2860 (-C-H_st_), 1258 (-OCH3st), 1100 (C-F); ^1^H NMR, δ (ppm) in DMSO-*d*_6_: 3.72 (s, 3H, -OC**H**_3_), 5.92 (s, 1H,=C**H**-_Pyrimidine_), 6.91–7.91 (m, 4H,=C**H**-_Phenyl_), 4.02 (br, 2H, -N**H**_2_), 9.02 (br, 2H, =N**H**-N**H**-), 10.20 (br, 1H, -N**H**-CS); ^13^C NMR, δ (ppm) in DMSO-*d*_6_: 52.9 (1C, -O**C**H_3_), 70.4 (1C, **-**C=**C**H-_Pyrimidine_), 112.0, 113.6, 129.7, 130.4 (5C, =**C**-, =**C**H-_Phenyl_), 159.3 (1C, =**C**-F_Phenyl_), 163.1 (1C, =**C**-NH_2 Pyrimidine_), 165.2 (1C, =**C**-NH-N=_Pyrimidine_), 171.1 (1C, =**C**-OCH_3 Pyrimidine_), 181.4 (1C, -**C**=S); MS (*m*/*z*): 308.0 (M^+^, 100.0%); Anal. calcd. For C_12_H_13_FN_6_OS (MW: 308.33); C, 46.74; H, 4.25; N, 27.26; S, 10.40; F, 6.16%; found: C, 46.77; H, 4.24; N, 27.27; S, 10.41; F, 6.17%.

##### 2-(2-Amino-6-Methoxypyrimidin-4-yl)-N-(4-Fluorophenyl)Hydrazine Carbothioamide (**5**)

Pink powder; yield (53.56%); m.p.: 180–181 °C; FT-IR (KBr, ν, cm^−1^): 3177, 3300 (N-H_st_), 1428 (C-N), 784 (C=S), 3490, 3334 (-N-H_st_), 1587 (-N-H_bend_); 1073 (C-N_st_Ar), 3029, 3060 (=C-H_st_), 2960, 2862 (-C-H_st_), 1254 (-OCH3st), 1100 (C-F); ^1^H NMR, δ (ppm) in DMSO-*d*_6_: 3.73 (s, 3H, -OC**H**_3_), 5.75 (s, 1H, =C**H**-_Pyrimidine_), 7.07–7.67 (m, 4H, =C**H**-_Phenyl_), 4.41 (br, 2H, -N**H**_2_), 8.80 (br, 2H, =N**H**-N**H**-), 9.77 (br, 1H, -N**H**-CS); ^13^C NMR, δ(ppm) in DMSO-*d*_6_: 53.1 (1C, -O**C**H_3_), 71.0 (1C, **-**C=**C**H-_Pyrimidine_), 115.0, 128.0, 136.0 (5C, =**C**-, =**C**H-_Phenyl_), 158.8 (1C, =**C**-F _Phenyl_), 160.8 (1C, =**C**-NH_2 Pyrimidine_), 163.2 (1C, =**C**-NH-N=_Pyrimidine_); 166.3 (1C, =**C**-OCH_3 Pyrimidine_), 181.0 (1C, -**C**=S); MS (*m*/*z*): 308.0 (M^+^, 100.0%); Anal. calcd. for C_12_H_13_FN_6_OS (MW: 308.33); C, 46.74; H, 4.25; N, 27.26; S, 10.40; F, 6.16%; found: C, 46.77; H, 4.24; N, 27.27; S, 10.41; F, 6.17%.

##### 2-(2-Amino-6-methoxypyrimidin-4-yl)-N-(3-chlorophenyl) Hydrazine Carbothioamide (**6**)

White powder; yield (70.09%); m.p.: 180.5–182 °C; FT-IR (KBr, ν, cm^−1^): 3177, 3299 (N-H_st_), 1429 (C-N), 783 (C=S), 3498, 3344 (-N-H_st_),1589 (-N-H_bend_),1074 (C-N_stAr_), 3029, 3060 (=C-H_st_), 2960, 2862 (-C-H_st_), 1260 (-OCH3st),700 (-Cl); ^1^H NMR, δ (ppm) in DMSO-*d*_6_: 3.73 (s, 3H, -OC**H**_3_), 6.25 (s, 1H, =C**H**-_Pyrimidine_), 7.15–8.08 (m, 4H, =C**H**-_Phenyl_), 5.15 (br, 2H, -N**H**_2_), 9.55 (br, 2H, =N**H**-N**H**-), 10.21 (br, 1H, -N**H**-CS); ^13^C NMR, δ (ppm) in DMSO-*d*_6_: 53.2 (1C, -O**C**H_3_), 70.0 (1C, -C=**C**H-_Pyrimidine_), 116.80, 124.0, 126.3, 129.8 (5C, =**C**-, =**C**H-_Phenyl_), 133.1 (1C, =**C**-Cl _Phenyl_), 141.3 (1C, =**C**-NH_2 Pyrimidine_), 158.0 (1C, =**C**-NH-N=_Pyrimidine_), 164.3 (1C, =**C**-OCH_3 Pyrimidine_), 181.2 (1C, -**C**=S); MS (*m*/*z*): 324.0 (M^+^, 79.93%); Anal. calcd. for C_12_H_13_ClN_6_OS (MW: 324.79); C, 44.38; H, 4.03; N, 25.88; S, 9.87; Cl, 10.92%; found: C, 44.40; H, 4.01; N, 25.87; S, 9.88; Cl, 10.93%.

##### 2-(2-Amino-6-methoxypyrimidin-4-yl)-N-(4-chlorophenyl)-hydrazine Carbothioamide (**7**)

Beige powder; yield (52.51%); m.p.: 199–200 °C; FT-IR (KBr, ν, cm^−1^): 3170, 3301 (N-H_st_), 1430 (C-N); 785 (C=S), 3488, 3341 (-N-H_st_), 1583 (-N-H_bend_), 1050 (C-N_stAr_), 3027, 3068 (=C-H_st_), 2941, 2864 (-C-H_st_), 1258 (-OCH3st), 677 (-Cl); ^1^H NMR, δ (ppm) in DMSO-*d*_6_: 3.73 (s, 3H, -OC**H**_3_), 5.39 (s, 1H,=C**H**-_Pyrimidine_), 7.13–7.70 (m, 4H,=C**H**-_Phenyl_), 4.44 (br, 2H, -N**H**_2_), 8.59 (br, 2H, =N**H**-N**H**-), 9.75 (br, 1H, -N**H**-CS); ^13^C NMR, δ (ppm) in DMSO-*d*_6_: 53.1 (1C, -O**C**H_3_), 70.70 (1C, -C=**C**H-,_Pyrimidine_), 126.1, 128.2 (5C, =**C**-, =**C**H-_Phenyl_), 147.4 (1C, =**C**-Cl_, Phenyl_), 160.1 (1C, =**C**-NH_2 Pyrimidine_), 163.2 (1C, =**C**-NH-N=_Pyrimidine_), 165.9 (1C, =**C**-OCH_3 Pyrimidine_), 186.0 (1C, -**C**=S); MS (*m*/*z*): 324.0 (M^+^, 100.0%); Anal. calcd. for C_12_H_13_ClN_6_OS (MW: 324.79); C, 44.38; H, 4.03; N, 25.88; S, 9.87; Cl, 10.92%; found: C, 44.40; H, 4.01; N, 25.87; S, 9.88; Cl, 10.93%.

##### 2-(2-Amino-6-methoxypyrimidin-4-yl)-N-(2-bromophenyl)-hydrazine Carbothioamide (**8**)

White powder; yield (69.22%); m.p.: 210–211 °C; FT-IR (KBr, ν, cm^−1^): 3109, 3300 (N-H_st_), 1428 (C-N), 784 (C=S), 3490, 3350 (-N-H_st_), 1587 (-N-H_bend_), 1073 (C-N_stAr_), 3029, 3060 (=C-H_st_), 2983, 2882 (-C-H_st_), 1254 (-OCH3st), 510 (-Br); ^1^H NMR, δ (ppm) in DMSO-*d*_6_: 3.73 (s, 3H, -OC**H**_3_), 5.35 (s, 1H, =C**H**-_Pyrimidine_), 7.7.08–7.81 (m, 4H, =C**H**-_Phenyl_), 4.90 (br, 2H, -N**H**_2_), 9.49 (br, 2H, =N**H**-N**H**-), 9.88 (br, 1H, -N**H**-CS); ^13^C NMR, δ (ppm) in DMSO-*d*_6_: 56.0 (1C, -O**C**H_3_), 73.6 (1C, -C=**C**H-_Pyrimidine_), 120.7 (1C, =**C**-Br _Phenyl_), 127.6, 129.0, 132.5, 138.1 (5C, =**C**-, =**C**H-_Phenyl_), 158.0 (1C, =**C**-NH_2 Pyrimidine_), 163.4 (1C, =**C**-NH-N=_Pyrimidine_), 164.2 (1C, =**C**-OCH_3 Pyrimidine_), 181.3 (1C, -**C**=S); MS (*m*/*z*): 369.0 (M^+^, 100.0%); Anal. calcd. for C_12_H_13_BrN_6_OS (MW: 369.24); C, 39.03; H, 3.55; N, 22.76; S, 8.68; Br, 21.64%; found: C, 39.05; H, 3.57; N, 22.75; S, 8.66; Br, 21.66%.

##### 2-(2-Amino-6-methoxypyrimidin-4-yl)-N-(3-bromophenyl)-hydrazine Carbothioamide (**9**)

White powder; yield (66.72%); m.p.: 187.5–188 °C; FT-IR (KBr, ν, cm^−1^): 3167, 3300 (N-H_st_), 1427 (C-N), 784 (C=S), 3490, 3350 (-N-H_st_), 1588 (-N-H_bend_), 1053 (C-N_st Ar_), 3029, 3060 (=C-H_st_), 2983, 2862 (-C-H_st_), 1254 (-OCH3st), 510 (-Br); ^1^H NMR, δ (ppm) in DMSO-*d*_6_: 3.73 (s, 3H, -OC**H**_3_), 5.25 (s, 1H, =C**H**-_Pyrimidine_), 6.78–7.77 (m, 4H, =C**H**-_Phenyl_), 4.86 (br, 2H, -N**H**_2_), 9.03 (br, 2H, =N**H**-N**H**-), 10.23 (br, 1H, -N**H**-CS); ^13^C NMR, δ (ppm) in DMSO-*d*_6_: 53.2 (1C, -O**C**H_3_), 70.3 (1C, -C=**C**H-_Pyrimidine_), 120.7 (1C, =**C**-Br _Phenyl_), 124.4, 127.7, 130.2, 131.0 (5C, =**C**-, =**C**H-_Phenyl_), 158.7 (1C, =**C**-NH_2 Pyrimidine_), 165.0 (1C, =**C**-NH-N=_Pyrimidine_), 171.1 (1C, =**C-**OCH_3 Pyrimidine_), 181.0 (1C, -**C**=S); MS (*m*/*z*): 369.0 (M^+^, 100.0%); Anal. calcd. for C_12_H_13_BrN_6_OS (MW: 369.24); C, 39.03; H, 3.55; N, 22.76; S, 8.68; Br, 21.64%; found: C, 39.05; H, 3.57; N, 22.75; S, 8.66; Br, 21.66%.

##### 2-(2-Amino-6-methoxypyrimidin-4-yl)-N-(4-bromophenyl)-hydrazine Carbothioamide (**10**)

Beige powder; yield (36.57%); m.p.: 189–189.5 °C; FT-IR (KBr, ν, cm^−1^): 3177, 3300 (N-H_st_), 1431 (C-N), 784 (C=S), 3490, 3334 (-N-H_st_), 1583 (-N-H_bend_), 1070 (C-N_stAr_), 3029, 3060 (=C-H_st_), 2940, 2862 (-C-H_st_), 1267 (-OCH3st), 523 (-Br); ^1^H NMR, δ (ppm) in DMSO-*d*_6_: 3.73 (s, 3H, -OC**H**_3_), 5.39 (s, 1H, =C**H**_Pyrimidine_), 7.24–7.87 (m, 4H, =C**H**-_Phenyl_), 4.49 (br, 2H, -N**H**_2_), 9.01 (br, 2H, =N**H**-N**H**-), 10.22 (br, 1H, -N**H**-CS); ^13^C NMR, δ (ppm) in DMSO-*d*_6_: 53.1 (1C, -O**C**H_3_), 70.8 (1C, -C=**C**H-_Pyrimidine_), 120.4 (1C, =**C**-Br,_Phenyl_), 128.0, 131.1 (5C, =**C**-, =**C**H-_Phenyl_), 160.1 (1C, =**C**-NH_2 Pyrimidine_), 163.2 (1C, =**C**-NH-N=_Pyrimidine_), 165.8 (1C, =**C**-OCH_3 Pyrimidine_), 171.0 (1C, -**C**=S); MS (*m*/*z*): 369.0 (M^+^, 100.0%); Anal. calcd. for C_12_H_13_BrN_6_OS (MW: 369.24); C, 39.03; H, 3.55; N, 22.76; S, 8.68; Br, 21.64%; found: C, 39.05; H, 3.57; N, 22.75; S, 8.66; Br, 21.66%.

##### 2-(2-Amino-6-methoxypyrimidin-4-yl)-N-(3-nitrophenyl)hydrazine Carbothioamide (**11**)

Yellow powder; yield (72.05%); m.p.: 173–174 °C; FT-IR (KBr, ν, cm^−1^): 3180, 3298 (N-H_st_),1426 (C-N), 780 (C=S), 3480, 3335 (-N-H_st_), 1580 (-N-H_bend_), 1072 (C-N_stAr_), 3025, 3058 (=C-H_st_), 2960, 2862 (-C-H_st_). 1254 (-OCH3st), 1518, 1327(-NO_2_); ^1^H NMR, δ (ppm) in DMSO-*d*_6_: 3.73 (s, 3H, -OC**H**_3_), 5.38 (s, 1H, =C**H**-_Pyrimidine_), 7.35–8.04 (m, 4H, =C**H**-_Phenyl_), 4.40 (br, 2H, -N**H**_2_), 8.85 (br, 3H, =N**H**-N**H**-), 10.42 (br, 1H, -N**H**-CS); ^13^C NMR, δ (ppm) in DMSO-*d*_6_: 53.1 (1C, -O**C**H_3_), 70.1 (1C, **-**C=**C**H-_Pyrimidine_), 111.7, 123.6, 129.1, 129.5 (5C, =**C**-, =**C**H-_Phenyl_), 148.6 (1C, =**C**-NO_2, Phenyl_), 162.9 (1C, =**C**-NH_2 Pyrimidine_), 164.6 (1C, =**C**-NH-N=_Pyrimidine_), 165.3 (1C, =**C**-OCH_3 Pyrimidine_); 171.4 (1C, -C=S); MS (*m*/*z*): 335.0 (M^+^, 100.0%); Anal. calcd. for C_12_H_13_N_7_O_3_S (MW: 335.34); C, 42.98; H, 3.91; N, 29.24; S, 9.56%; found: C, 42.99; H, 3.91; N, 29.20; S, 9.57%.

##### 2-(2-Amino-6-methoxypyrimidin-4-yl)-N-(4-nitrophenyl)hydrazine Carbothioamide (**12**)

Orange powder; yield (73.35%); m.p.: 179–180 °C; FT-IR (KBr, ν, cm^−1^): 3177, 3300 (N-H_st_), 1428 (C-N), 784 (C=S), 3486, 3334 (-N-H_st_), 1586 (-N-H_bend_), 1073 (C-N_stAr_), 3029, 3060 (=C-H_st_), 2960, 2861(-C-H_st_), 1254 (-OCH3st), 1518, 1327 (-NO_2_); ^1^H NMR, δ (ppm) in DMSO-*d*_6_: 3.73 (s, 3H, -OC**H**_3_), 5.39 (s, 1H, =C**H**-_Pyrimidine_), 7.13–8.16 (m, 4H, =C**H**-_Phenyl_), 4.42 (br, 2H, -N**H**_2_), 9.03 (br, 2H, =N**H**-N**H**-), 10.13 (br, 1H, -N**H**-CS); ^13^C NMR, δ (ppm) in DMSO-*d*_6_: 53.3 (1C, -O**C**H_3_), 70.1 (1C, -C=**C**H-_Pyrimidine_), 116.7, 124.0, 125.5 (5C, =**C**-, =**C**H-_Phenyl_), 148.6 (1C, =**C**-NO_2, Phenyl_), 153.3 (1C, =**C**-NH_2 Pyrimidine_), 164.6 (1C, =**C**-NH-N=_Pyrimidine_), 165.3 (1C, =**C**-OCH_3 Pyrimidine_), 171.4 (1C, -**C**=S); MS (*m*/*z*): 335.0 (M^+^, 52.25%); Anal. calcd. for C_12_H_13_N_7_O_3_S (MW: 335.34); C, 42.98; H, 3.91; N, 29.24; S, 9.56%; found: C, 42.99; H, 3.91; N, 29.20; S, 9.57%.

##### 2-(2-Amino-6-methoxypyrimidin-4-yl)-N-(2-methoxyphenyl)-hydrazine Carbothioamide (**13**)

Purple powder; yield (87.83%); m.p.: 169–170 °C; FT-IR (KBr, ν, cm^−1^): 3174, 3300 (N-H_st_), 1428 (C-N), 784 (C=S), 3490, 3334 (-N-H_st_), 1582 (-N-H_bend_), 1072 (C-N_stAr_), 3030, 3060 (=C-H_st_), 2962, 2862 (-C-H_st_), 1254 (-OCH3st); ^1^H NMR, δ (ppm) in DMSO-*d*_6_: 3.73 (s, 6H, -OC**H**_3_), 6.12 (s, 1H, =C**H**-_Pyrimidine_), 6.86–7.79 (m, 4H, =C**H**-_Phenyl_), 4.01 (br, 2H, -N**H**_2_), 8.62 (br, 2H, =N**H**-N**H**-), 9.78 (br, 1H, -N**H**-CS); ^13^C NMR, δ (ppm) in DMSO-*d*_6_: 53.2 (2C, -O**C**H_3_), 70.3 (1C, -C=**C**H-_Pyrimidine_), 116.3, 121.8, 124.4, 127.7 (5C, =**C**-, =**C**H-_Phenyl_), 155.8 (1C, =**C**-OCH_3_-_Phenyl_), 158.5 (1C, =**C**-NH_2 Pyrimidine_), 164.4 (1C, =**C**-NH-N=_Pyrimidine_), 171.2 (1C, =**C**-OCH_3 Pyrimidine_), 181.2 (1C, -**C**=S); MS (*m*/*z*): 320.0 (M^+^, 90.0%); Anal. calcd. for C_13_H_16_N_6_O_2_S (MW: 320.37); C, 48.74; H, 5.03; N, 26.23; S, 10.01%; found: C, 48.73; H, 5.01; N, 26.21; S, 10.00%.

##### 2-(2-Amino-6-methoxypyrimidin-4-yl)-N-(3-methoxyphenyl)-hydrazine Carbothioamide (**14**)

Purple powder; yield (73.85%); m.p.: 182.5–184 °C; FT-IR (KBr, ν, cm^−1^): 3177, 3243 (N-H_st_), 1428 (C-N), 782 (C=S), 3490, 3334 (-N-H_st_), 1585 (-N-H_bend_); 1073 (C-N_stAr_), 3029, 3060 (=C-H_st_), 2960, 2862 (-C-H_st_), 1250 (-OCH3st); ^1^H NMR, δ (ppm) in DMSO-*d*_6_: 3.73 (s, 6H, -OC**H**_3_), 5.95 (s, 1H, =C**H**-_Pyrimidine_), 7.16–8.90 (m, 4H, =C**H**-_Phenyl_), 4.68 (br, 2H, -N**H**_2_), 8.90, 8.42 (br, 2H, =N**H**-N**H**-), 9.67 (br, 1H, -N**H**-CS); ^13^C NMR, δ (ppm) in DMSO-*d*_6_: 55.5 (2C, -O**C**H_3_), 70.6 (1C, -C=**C**H-_Pyrimidine_), 110.8, 129.1 (5C, =**C**-, =**C**H _Phenyl_), 158.8 (1C, =**C**-OCH_3_-_Phenyl_), 159.4 (1C, =**C**-NH_2 Pyrimidine_), 163.1 (1C, =**C**-NH-N=_Pyrimidine_), 165.4 (1C, =**C**-OCH_3 Pyrimidine_), 181.0 (1C, -**C**=S); MS (*m*/*z*): 320.0 (M^+^, 100.0%); Anal. calcd. for C_13_H_16_N_6_O_2_S (MW: 320.37); C, 48.74; H, 5.03; N, 26.23; S, 10.01%; found: C, 48.73; H, 5.01; N, 26.21; S, 10.00%.

##### 2-(2-Amino-6-methoxypyrimidin-4-yl)-N-(4-methoxyphenyl) Hydrazine Carbothioamide (**15**)

Beige powder; yield (69.55%); m.p.: 209–210 °C; FT-IR (KBr, ν, cm^−1^): 3171, 3300 (N-H_st_), 1425 (C-N), 784 (C=S), 3490, 3334 (-N-H_st_),1582 (-N-H_bend_), 1070 (C-N_stAr_), 3025, 3058 (=C-H_st_), 2960, 2862 (-C-H_st_), 1250 (-OCH3st); ^1^H NMR, δ (ppm) in DMSO-*d*_6_: 3.73 (s, 6H, -OC**H**_3_), 5.39 (s, 1H, =C**H**-_Pyrimidine_), 6.83–7.25 (m, 4H, =C**H**-_Phenyl_), 4.36 (br, 2H, -N**H**_2_), 8.53 (br, 2H, =N**H**-N**H**-), 9.58 (br, 1H, -N**H**-CS); ^13^C NMR, δ (ppm) in DMSO-*d*_6_: 55.5 (2C, -O**C**H_3_), 70.6 (1C, -C=**C**H-_Pyrimidine_), 110.8, 117.4, 129.1 (5C, =**C**-, =**C**H-_Phenyl_), 140.9 (1C, =C-OCH_3_-_Phenyl_), 159.4 (1C, =**C**-NH_2 Pyrimidine_), 163.1 (1C, =**C**-NH-N=_Pyrimidine_), 165.4 (1C, =**C**-OCH_3 Pyrimidine_), 181.0 (1C, -**C**=S); MS (*m*/*z*): 320.0 (M^+^, 65.60%); Anal. calcd. for C_13_H_16_N_6_O_2_S (MW: 320.37); C, 48.74; H, 5.03; N, 26.23; S, 10.01%; found: C, 48.73; H, 5.01; N, 26.21; S, 10.00%.

##### 2-(2-Amino-6-methoxypyrimidin-4-yl)-N-(p-tolyl)hydrazine Carbothioamide (**16**)

Brown powder; yield (57.53%); m.p.: 198–199 °C; FT-IR (KBr, ν, cm^−1^): 3177, 3300 (N-H_st_), 1428 (C-N), 784 (C=S), 3490, 3334 (-N-H_st_), 1581 (-N-H_bend_), 1070 (C-N_st_Ar), 3029, 3060 (=C-H_st_), 2960, 2862 (-C-H_st_), 1266 (-OCH3st); ^1^H NMR, δ (ppm) in DMSO-*d*_6_: 1.81 (s, 3H, -C**H**_3_), 3.73 (s, 3H, -OC**H**_3_), 5.36 (s, 1H,=C**H-**_Pyrimidine_), 7.02–7.43 (m, 4H,=C**H**-_Phenyl_), 4.26 (br, 2H, **-**N**H**_2_), 8.53(br, 2H, =N**H**-N**H**-), 9.56 (br, 1H, -N**H**-CS); ^13^C NMR, δ (ppm) in DMSO-*d*_6_: 21.0 (1C, -**C**H_3_), 53.2 (1C, -O**C**H_3_), 71.1 (1C, -C=**C**H-_Pyrimidine_), 125.6, 128.9, 155.8 (5C, =**C**-, =**C**H-_Phenyl_), 147.3 (1C, =**C**-CH_3Phenyl_), 160.8 (1C, =**C**-NH_2 Pyrimidine_), 163.3 (1C, =**C**-NH-N=_Pyrimidine_), 166.3 (1C, =**C**-OCH_3 Pyrimidine_), 186.0 (1C, -**C**=S); MS (*m*/*z*): 304.0 (M^+^, 100.0%); Anal. calcd. for C_13_H_16_N_6_OS (MW: 304.37); C, 51.30; H, 5.30; N, 27.61; S, 10.53%; found: C, 50.31; H, 5.31; N, 27.61; S, 10.51%.

##### 2-(2-Amino-6-methoxypyrimidin-4-yl)-N-(3-cyanophenyl)hydrazine Carbothioamide (**17**)

Brown powder; yield (61.39%); m.p.: 174–175 °C; FT-IR (KBr, ν, cm^−1^): 3170, 3300 (N-H_st_), 1428 (C-N), 784 (C=S), 3490, 3334 (-N-H_st_), 1578 (-N-H_bend_), 1073 (C-N_stAr_), 3025, 3063 (=C-H_st_), 2962, 2866 (-C-H_st_), 1254 (-OCH3st), 2232, 1486 (-CN_st_); ^1^H NMR, δ (ppm) in DMSO-*d*_6_: 3.73 (s, 3H, -OC**H**_3_), 5.39 (s, 1H, =C**H**-_Pyrimidine_), 7.01–7.88 (m, 4H, =C**H**-_Phenyl_), 4.89, (br, 2H, -N**H**_2_), 8.67 (br, 2H, =N**H**-N**H**-), 9.94 (br, 1H,-N**H**-CS); ^13^C NMR, δ (ppm) in DMSO-*d*_6_: 53.3 (1C, -O**C**H_3_), 70.2 (1C, -C=**C**H-_Pyrimidine_), 111.3 (1C, -**C**-CN_, Phenyl_), 122.1 (1C, -**C**N), 128.6, 129.9, 130.4 (5C, =**C**-, =**C**H-_Phenyl_), 143.9 (1C, =**C**-NH_2 Pyrimidine_), 158.7 (1C, =**C**-NH-N=_Pyrimidine_), 164.9 (1C, =**C**-OCH_3 Pyrimidine_), 186.0 (1C, -C=S); MS (*m*/*z*): 315.0 (M^+^, 100.0%); Anal. calcd. for C_13_H_13_N_7_OS (MW: 315.35); C, 49.51; H, 4.16; N,31.09; S, 10.17%; found: C, 49.52; H, 4.16; N, 31.12; S, 10.19%.

### 3.3. Biological Activity as Antidiabetic Activity of Most of Synthetic Compound of Pyrimidine Derivatives

#### 3.3.1. α-Amylase Activity Assay

The α-amylase activity assay was performed according to our previous reported work [31] by mixing samples (50 μL) and the substrate solution (50 μL), adding an assay buffer (50 μL) and reaction mixture (50 μL) to each well, and then measuring absorbance at room temperature, against acarbose as the standard drug. The % inhibition was calculated as follows:

% _Inhibition_ = (Absorbance_control_ − Absorbance_compound_) × 100/Absorbance_control_

#### 3.3.2. α-Glucosidase Activity Assay

The *α*-glucosidase activity assay was conducted as we reported earlier [31]. Samples (50 μL) were added to 96 wells, and the volume was adjusted to 100 μL/well with an assay buffer. For the reaction, 50 μL of reaction mix for each reaction were performed by adding assay buffer 47 μL and substrate mix 3 μL. An amount of 50 μL of the reaction mix was added into each well, it was mixed thoroughly, and the absorbance was recorded at room temperature, against acarbose as the standard drug. 

### 3.4. Computational Simulation Studies

The computational studies were carried out using the Maestro module (version 11.8) of Schrodinger software suite (Schrodinger, LLC., New York, NY, USA, 2018) [53].

#### 3.4.1. Crystal Structures

The X-ray coordinates of α-glucosidase and α-amylase (PDB IDs: 3W37 and 4GQR, respectively) were obtained from the Research Collaboratory for Structural Bioinformatics (RCSB) Protein Data Bank (PDB) [54].

#### 3.4.2. Protein Preparation

The PDB structures were prepared for docking using the Protein Preparation Workflow. The preparation and minimization process were carried out at a pH of 7.4, and ionization states were adjusted. Polar hydrogens were added, and non-essential water molecules were excluded from the structures. The targets’ structures were finally minimized by using an OPLS3 force field with a default value for rmsd of 0.30 Å for non-hydrogen atoms [55].

### 3.5. Receptor Grid Generation

The receptor grids were positioned at the center of the bound ligand for each receptor with a 1.00 van der Waals radius along with a cutoff of 0.25 for partial charges. A grid box of 20 Å^3^ was generated for the binding site using default parameters and without any constraints.

#### 3.5.1. Ligand Library Preparation

The ligands to be docked were prepared and optimized for docking using LigPrep by generating the most likely ionization states at a pH of 7 ± 1 while keeping the original ionization state intact. During this process, the ligands’ structures were optimized with the OPLS3 force field. The produced ligands’ conformations were used for docking without further modifications.

#### 3.5.2. Validation of Molecular Docking

The validation of the molecular docking was performed by calculating the accuracy of the predicted conformations to match the experimental conformations [56,57,58]. The crystallographic ligands were docked into their respective targets using the same docking criteria used for the prepared ligands. The docked position with the lowest binding energy was then aligned with the conformation of the crystallographic structure using Maestro’s structure superimposition tool, and the root mean square deviation (RMSD) of the alignments was then calculated.

#### 3.5.3. Molecular Docking

The extra precision mode (XP) on glide was used for all docking procedures with a van der Waals (vdw) radius scaling factor of 0.80 and a partial charge cut-off of 0.15 and with no constraints. The XP score was utilized to rank ligands and determine the optimal docked pose for each ligand. Molecular mechanics–generalized Born surface area (MM-GBSA) binding free energy was computed for the top ranked poses using the Prime MM-GBSA module and put in context with the experimental binding affinities. The energy was calculated based on the solvent accessible area in comparison to the energy of individual molecules by calculating the difference in energy of the individual ligand and receptor and the energy of the receptor-ligand complex. Visual evaluation of key binding interactions was also used to finally assess the best active compounds and develop the structure activity relationship. Figures were generated using PyMol 2.0.6 Graphical Software (Schrödinger^®^, New York, NY, USA).

## 4. Conclusions

2-Amino-6-methoxypyrimidine derivatives (**2**–**17**) were synthesized and characterized successfully. The synthesized derivatives were tested against α-glucosidase and α-amylase enzymes. The derivative **4** exhibited the highest activity against α-glucosidase and α-amylase enzymes with IC_50_ 12.16 ±0.12 µM and IC_50_ 11.13 ± 0.12 µM, respectively. It could be ascribed to the presence of more electronegative fluorine atoms and their suitable interaction with the enzymes. The docking studies were in agreement with the experimental inhibition activity and identified the key structural characteristics of the 2,4-diaminopyrimidine scaffold necessary for the dual inhibition of α-glucosidase and α-amylase.

## Figures and Tables

**Table 1:** The biological activities of the pyrimidine derivatives against *α*-glucosidase and α-amylase (IC_50_ “μM” ± SD, *n* = 5) for selected compounds.

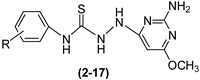			
**Comp. No**	**R**	***α*** **-Glucosidase (IC_50_, µM)**	**α** **-Amylase (IC_50_, µM)**
**4**	3-F	12.16 ± 0.12	11.13 ± 0.12
**6**	3-Cl	16.13 ± 0.11	15.17 ± 0.07
**9**	3-Br	19.11 ± 0.11	18.16 ± 0.08
**11**	3-NO_2_	22.11 ± 0.13	21.14 ± 0.12
**14**	3-OCH_3_	30.12 ± 0.13	33.18 ± 0.14
**16**	4-CH_3_	36.14 ± 0.12	34.24 ± 0.16
**17**	3-CN	42.19 ± 0.13	40.16 ± 0.11
Acarbose		10.60 ± 0.17	11.30 ± 0.12

**Table 2:** Experimental IC_50_, XP score, and computational binding energy of the pyrimidine derivatives and the reference inhibitor, acarbose, with *α*-glucosidase and α-amylase.

Comp. No	*α*-Glucosidase			α-Amylase		
	(IC_50_, µM)	XP Score	MM-GBSA dG Bind	(IC_50_, µM)	XP Score	MM-GBSA dG Bind
**4**	12.16 ± 0.12	−4.1	5.3	11.13 ± 0.12	−4.5	−0.4
**6**	16.13 ± 0.11	−5.0	13.6	15.17 ± 0.07	−4.7	−8.8
**9**	19.11 ± 0.11	−4.8	12.7	18.16 ± 0.08	−5.0	0.4
**11**	22.11 ± 0.13	−3.9	29.2	21.14 ± 0.12	−4.8	−4.1
**14**	30.12 ± 0.13	−2.9	17.3	33.18 ± 0.14	−5.2	11.5
**16**	36.14 ± 0.12	−4.8	−2.7	34.24 ± 0.16	−4.3	−6.5
**17**	42.19 ± 0.13	−2.4	16.0	40.16 ± 0.11	−4.3	−2.7
Acarbose	10.60 ± 0.17	−11.5	−29.9	11.30 ± 0.12	−10.0	−34.7

**Table 3:** Relative mean standard deviation (RMSD) values of the binding poses of compounds **4**, **6, 9**, **11**, **14**, **16**, and **17** in reference to compound **4**.

Comp. No	*α*-Glucosidase		α-Amylase	
	(IC_50_, µM)	RMSD	(IC_50_, µM)	RMSD
**4**	12.16 ± 0.12	0.0	11.13 ± 0.12	0.0
**6**	16.13 ± 0.11	0.07	15.17 ± 0.07	0.6
**9**	19.11 ± 0.11	1.5	18.16 ± 0.08	1.7
**11**	22.11 ± 0.13	1.7	21.14 ± 0.12	1.7
**14**	30.12 ± 0.13	2.3	33.18 ± 0.14	1.8
**16**	36.14 ± 0.12	1.2	34.24 ± 0.16	1.2
**17**	42.19 ± 0.13	8.6	40.16 ± 0.11	6.3

## Data Availability

In silico drug experiments using molecular docking to target α-glucosidase and α-amylase (PDB IDs: 3W37, and 4GQR, respectively) were obtained from the Research Collaboratory for Structural Bioinformatics (RCSB) Protein Data Bank (PDB). α-glucosidase: https://doi.org/10.2210/pdb3W37/pdb; α-amylase: https://doi.org/10.2210/pdb4GQR/pdb; Other data are contained within the article and Supplementary Materials.

## Supplementary Materials

The following supporting information can be downloaded at https://www.mdpi.com/article/10.3390/molecules30132857/s1.
