# Supplementary material for: One-Pot Synthesis of Novel Pyrimidine Derivatives with Potential Antidiabetic Activity Through Dual α-Glucosidase and α-Amylase Inhibitors
Source: Molecules. 2025 Jul 4;30(13):2857. doi: 10.3390/molecules30132857 (PMC12251307; doi:10.3390/molecules30132857)
Supplement: Supplementary file 1 [file molecules-30-02857-s001.zip › molecules-3693294-supplementary.pdf]

Supplementary

Title:

One-Pot Synthesis of Novel Pyrimidine Derivatives with

Potential Antidiabetic Activity Through Dual α-Glucosidase and α-Amylase Inhibitors

Authors:

Ohood Al-Shehri, Samar Abubshait *, Muhammad Nawaz, Mohamed S. Gomaa and Haya A. Abubshait

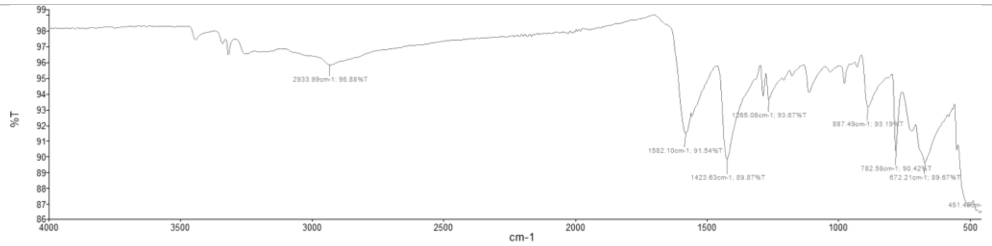

Figure S1. FTIR spectrum of compound (1)

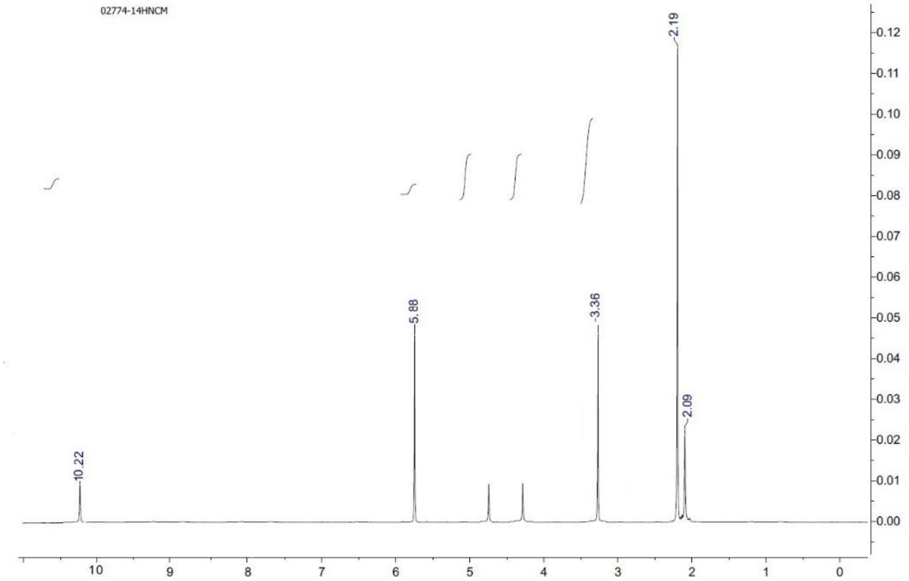

Figure S2. ^{1}H NMR spectrum of compound (1)

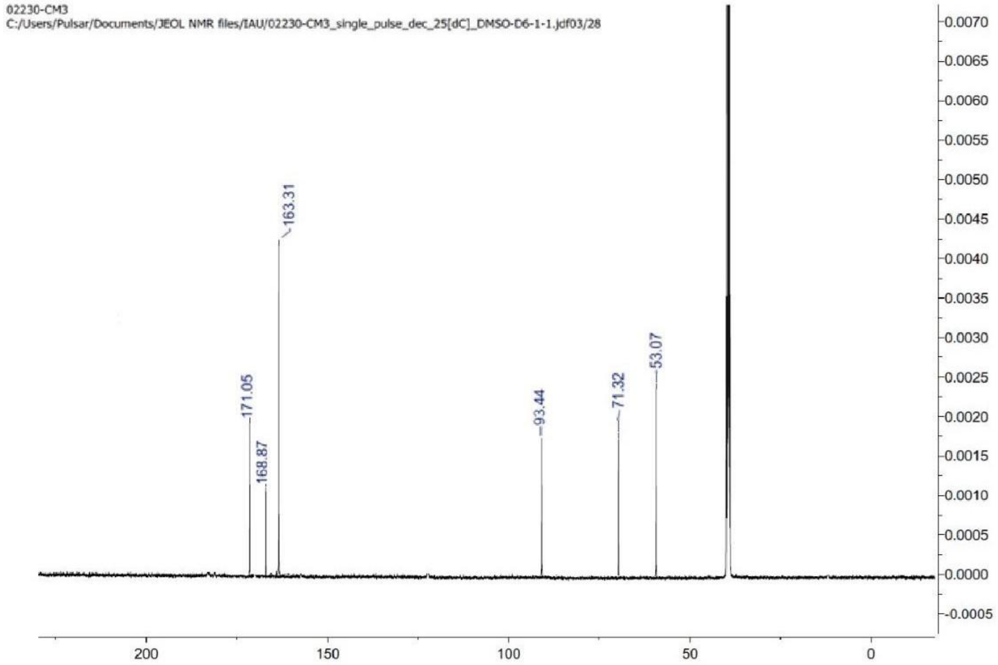

Figure S3. ^{13}C NMR spectrum of compound (1)

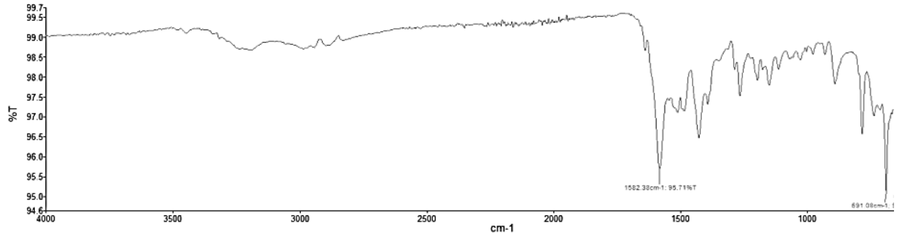

Figure S4. FTIR spectrum of compound (2)

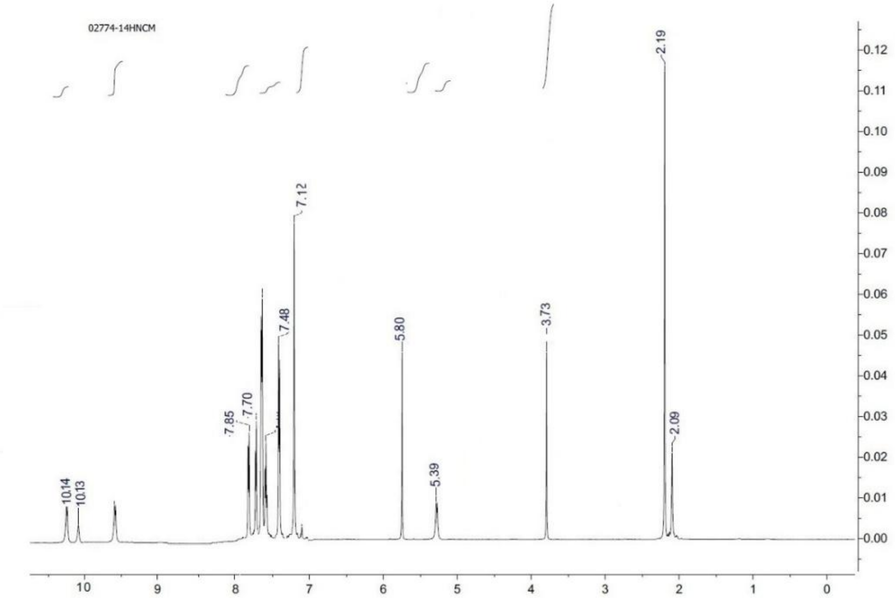

Figure S5. ^{1}H NMR spectrum of compound (2)

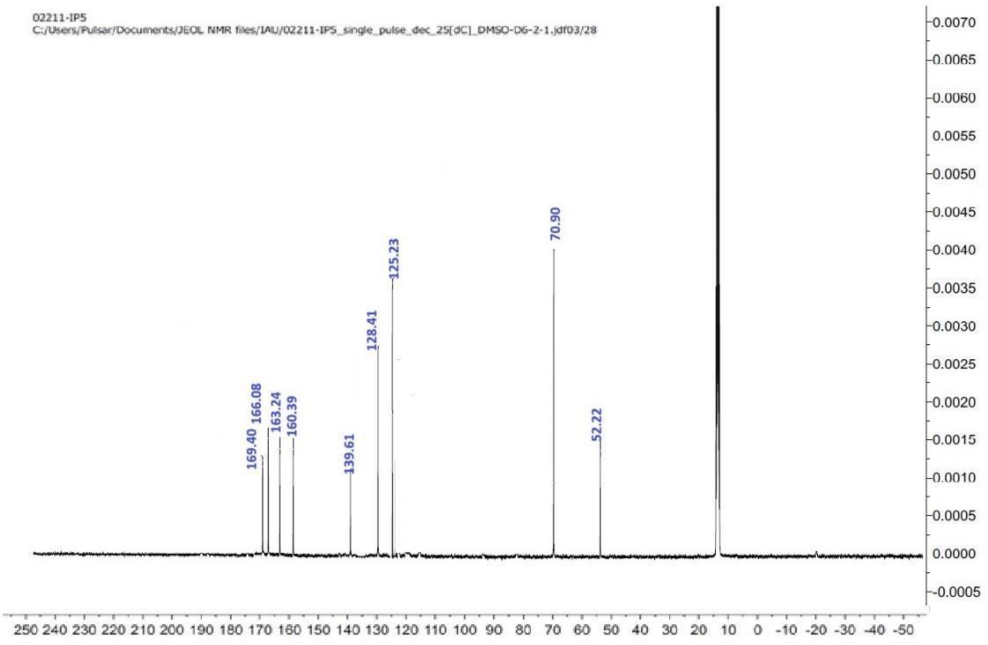

Figure S6. ^{13}C NMR spectrum of compound (2)

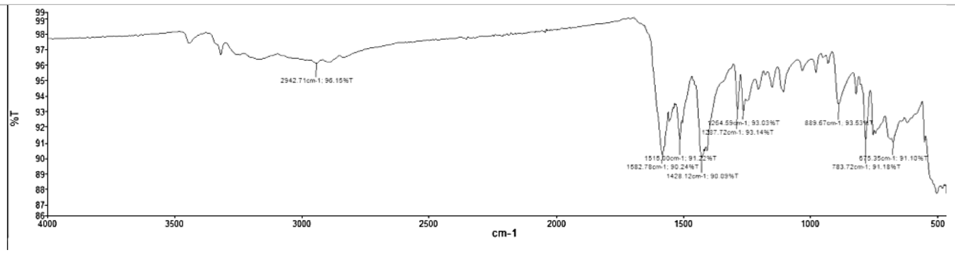

Figure S7. FTIR spectrum of compound (3)

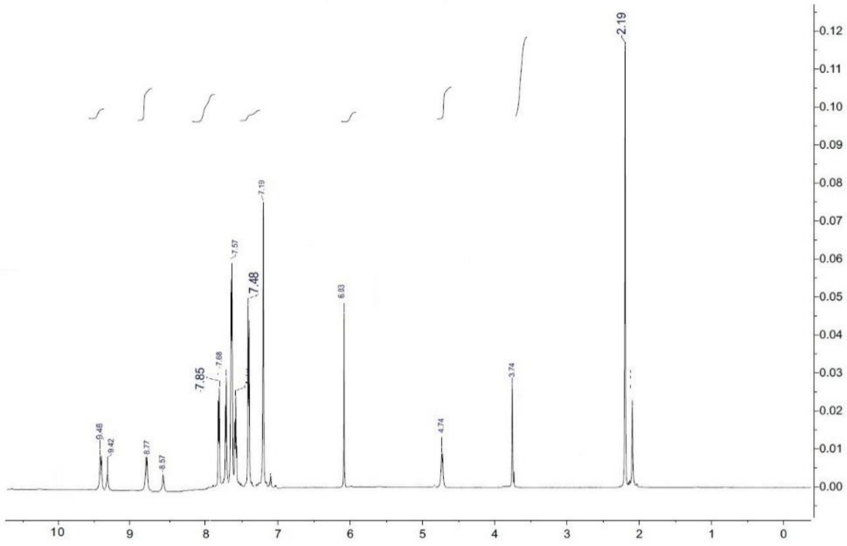

Figure S8. ^{1}H NMR spectrum of compound (3)

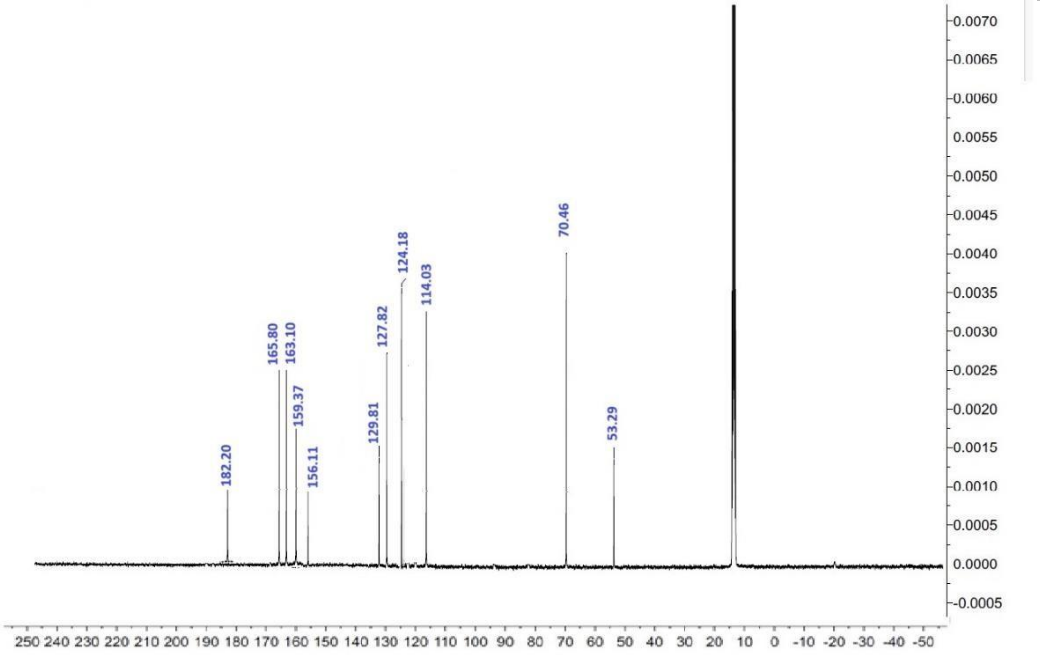

Figure S9. ^{13}C NMR spectrum of compound (3)

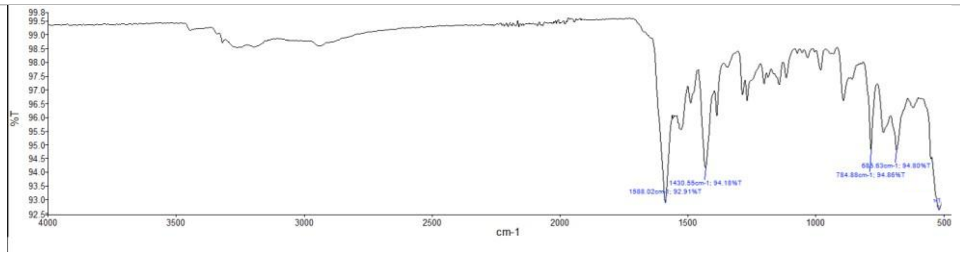

Figure S10. FTIR spectrum of compound (4)

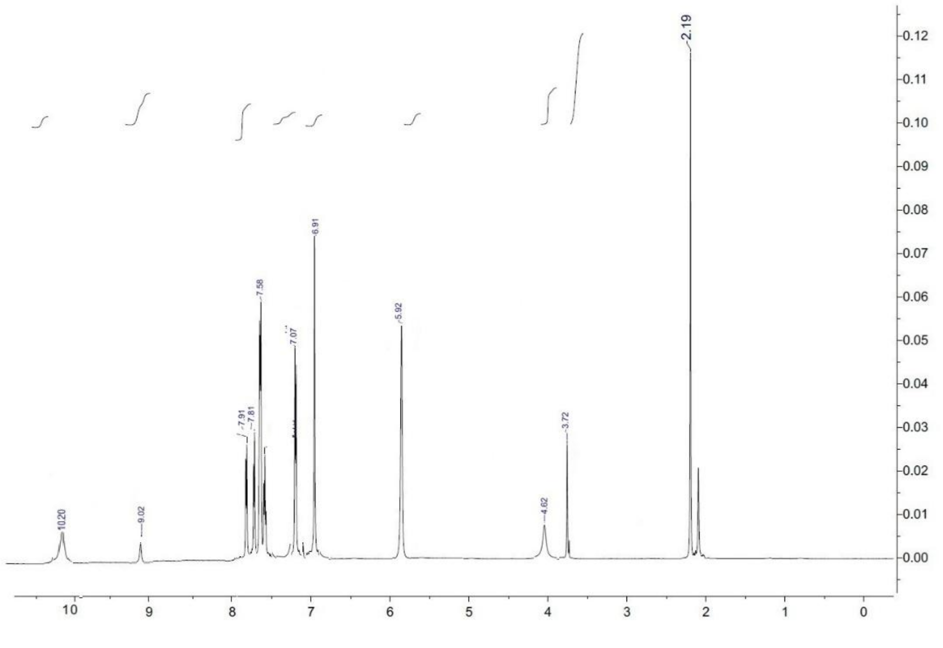

Figure S11. ^{1}H NMR spectrum of compound (4)

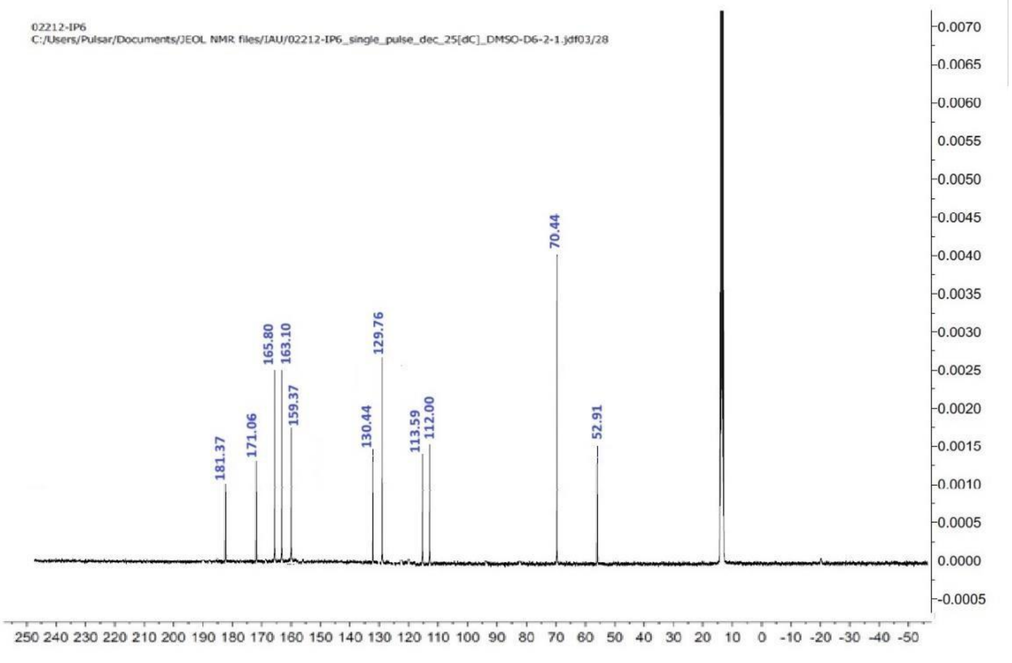

Figure S12. ^{13}C NMR spectrum of compound (4)

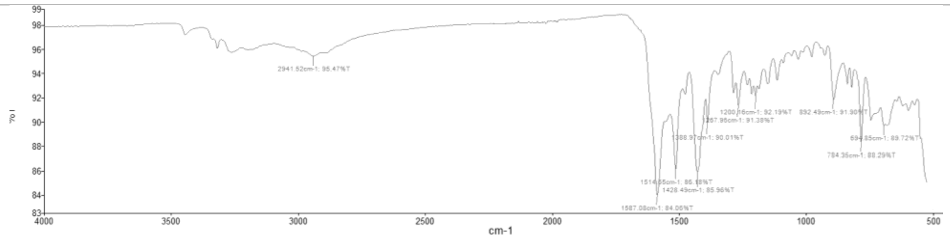

Figure S13. FTIR spectrum of compound (5)

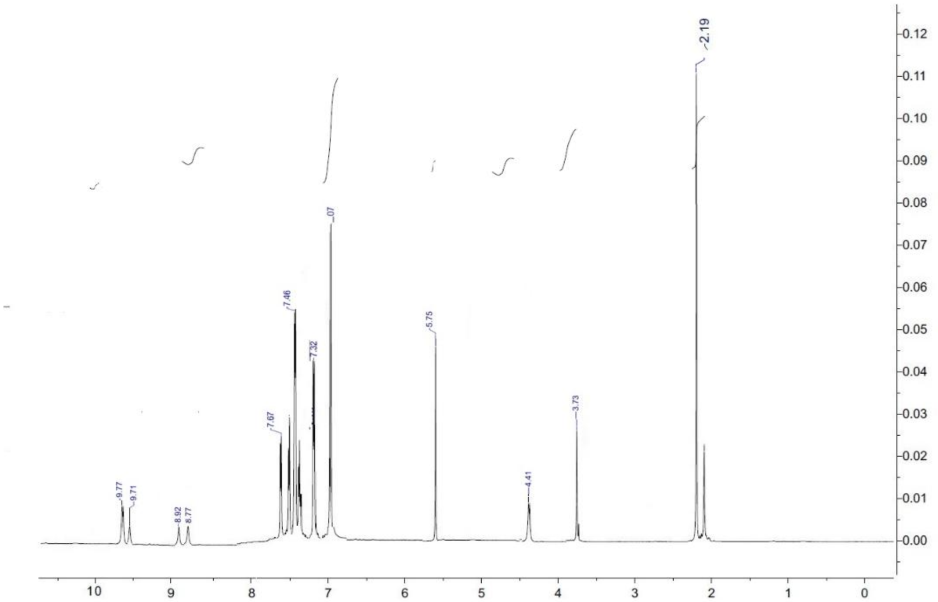

Figure S14. ^{1}H NMR spectrum of compound (5)

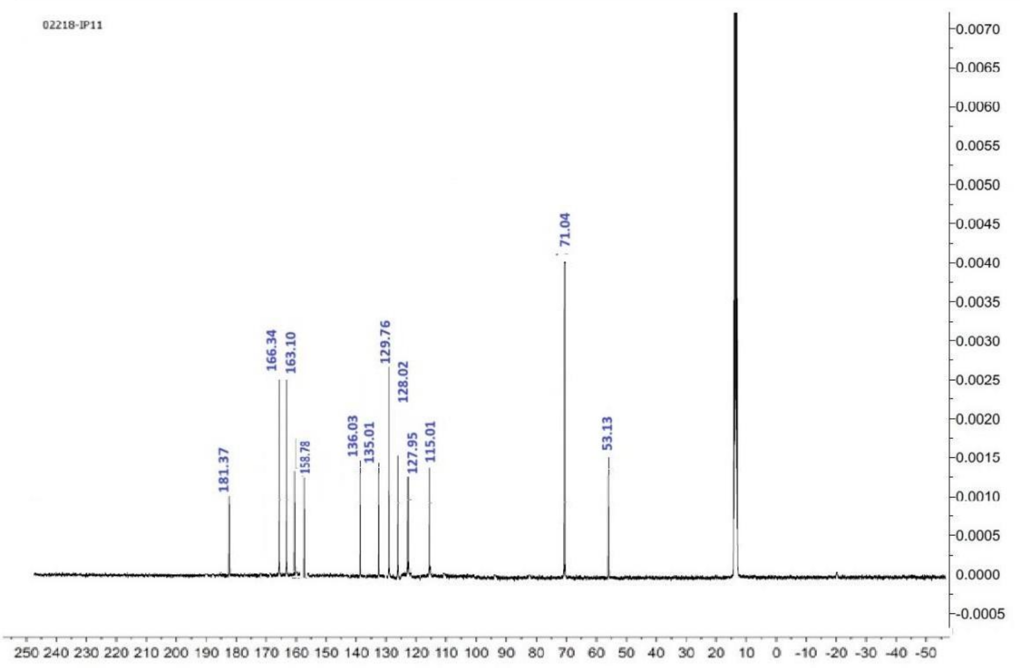

Figure S15. ^{13}C NMR spectrum of compound (5)

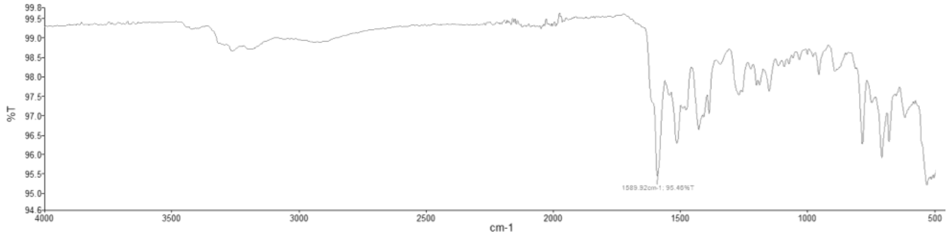

Figure S16. FTIR spectrum of compound (6)

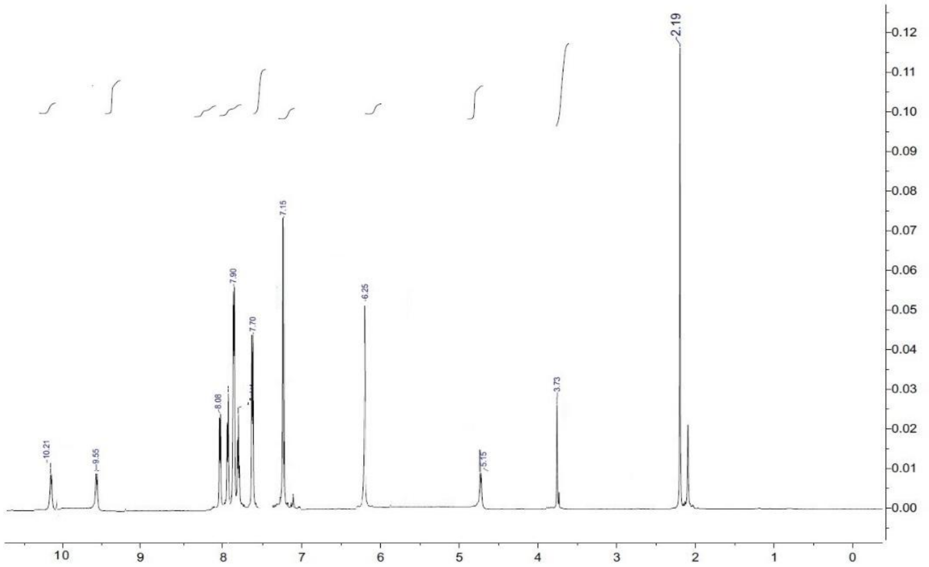

Figure S17. ^{1}H NMR spectrum of compound (6)

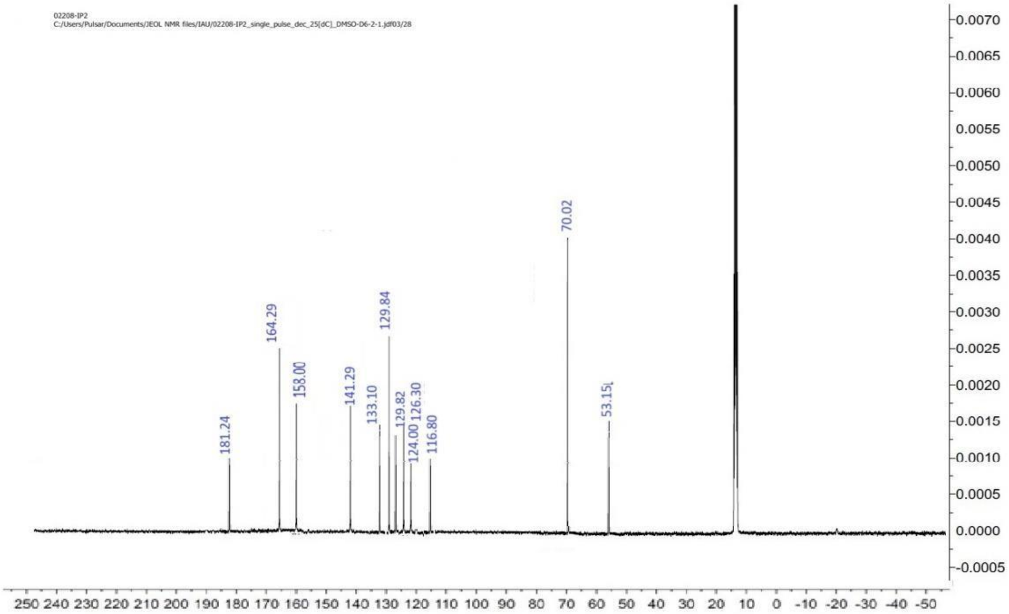

Figure S18. ^{13}C NMR spectrum of compound (6)

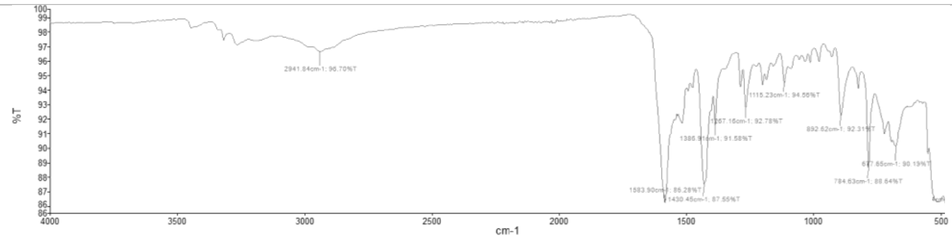

Figure S19. FTIR spectrum of compound (7)

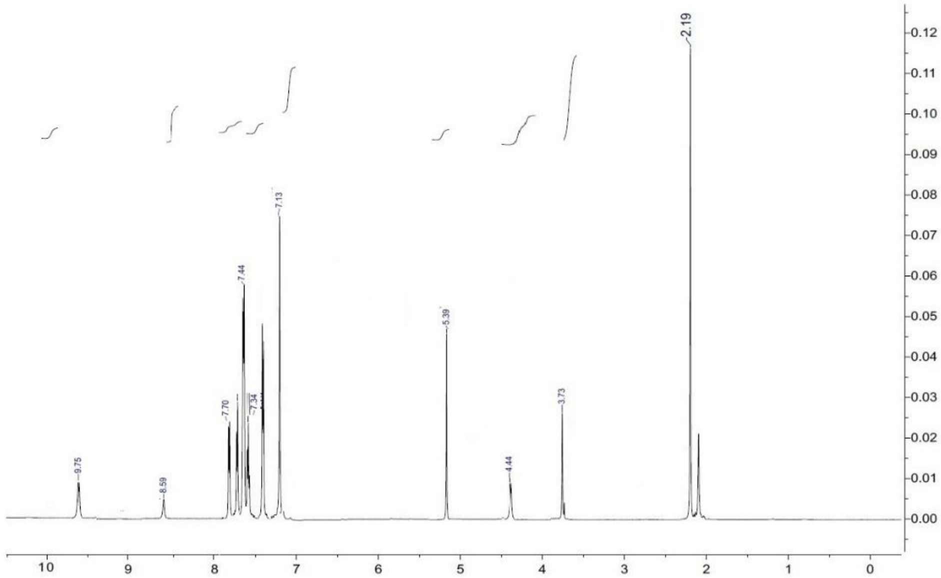

Figure S20. ^{1}H NMR spectrum of compound (7)

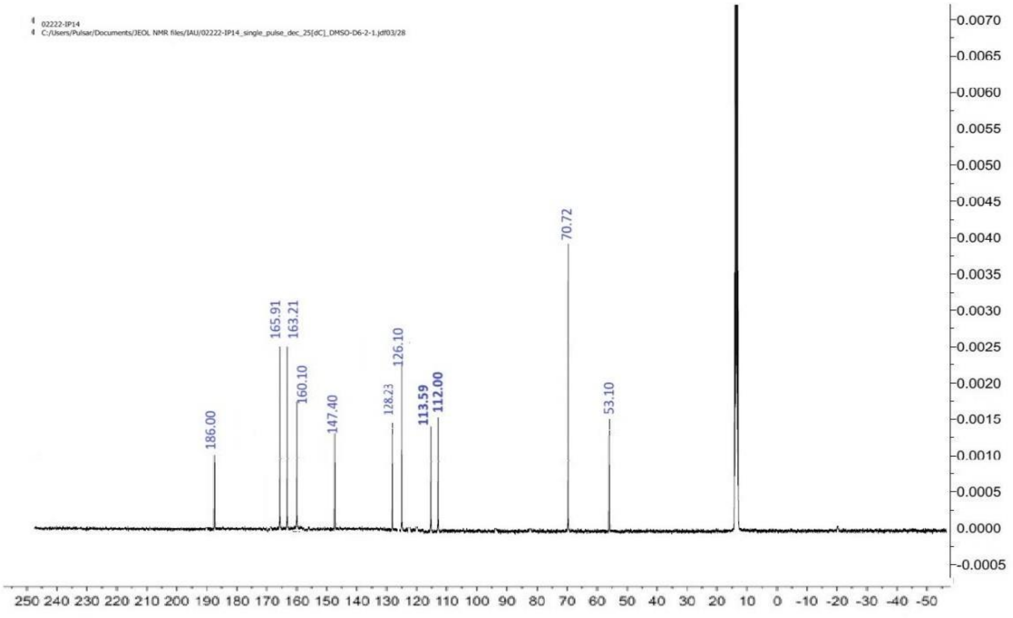

Figure S21. ^{13}C NMR spectrum of compound (7)

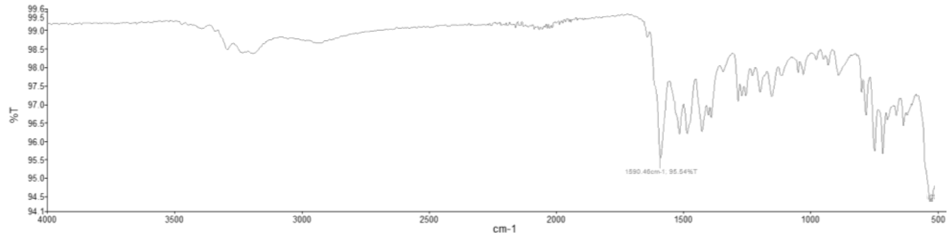

Figure S22. FTIR spectrum of compound (8)

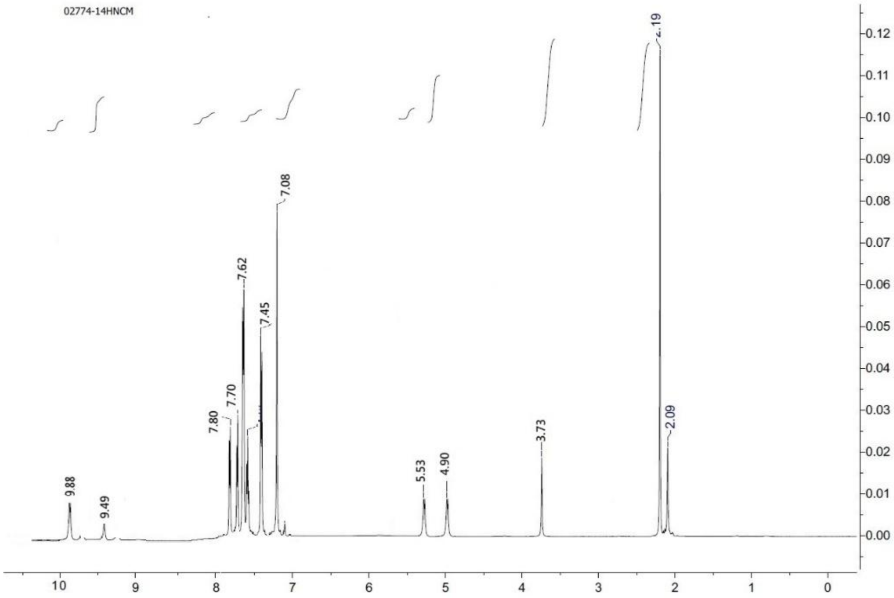

Figure S23. ^{1}H NMR spectrum of compound (8)

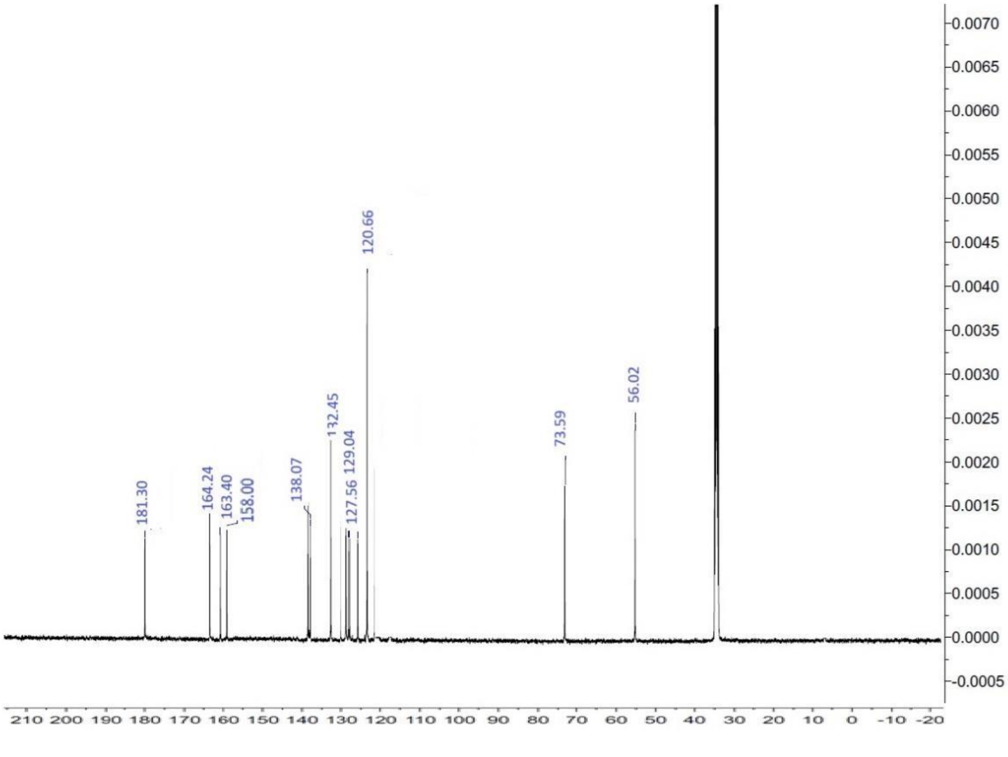

Figure S24. ^{13}C NMR spectrum of compound (8)

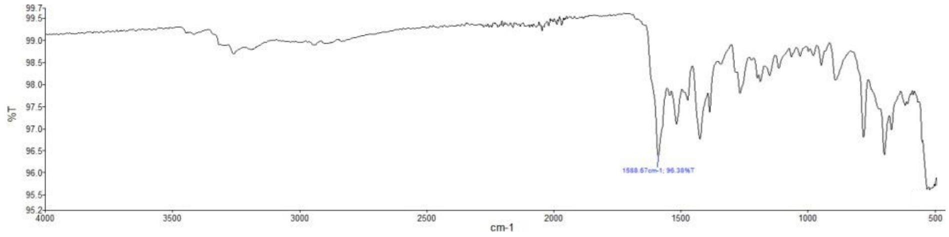

Figure S25. FTIR spectrum of compound (9)

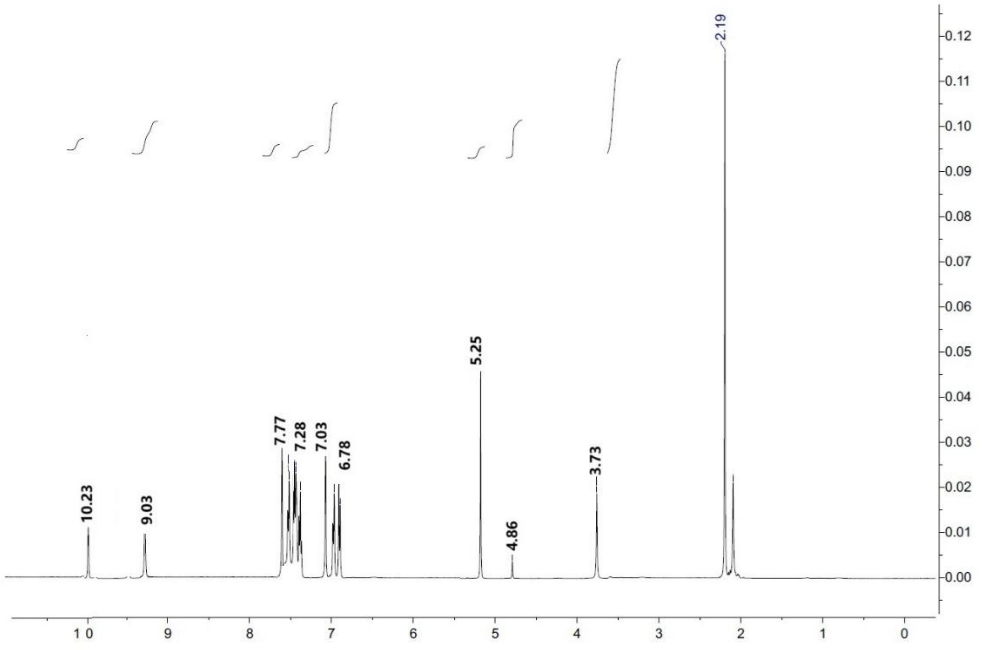

Figure S26. ^{1}H NMR spectrum of compound (9)

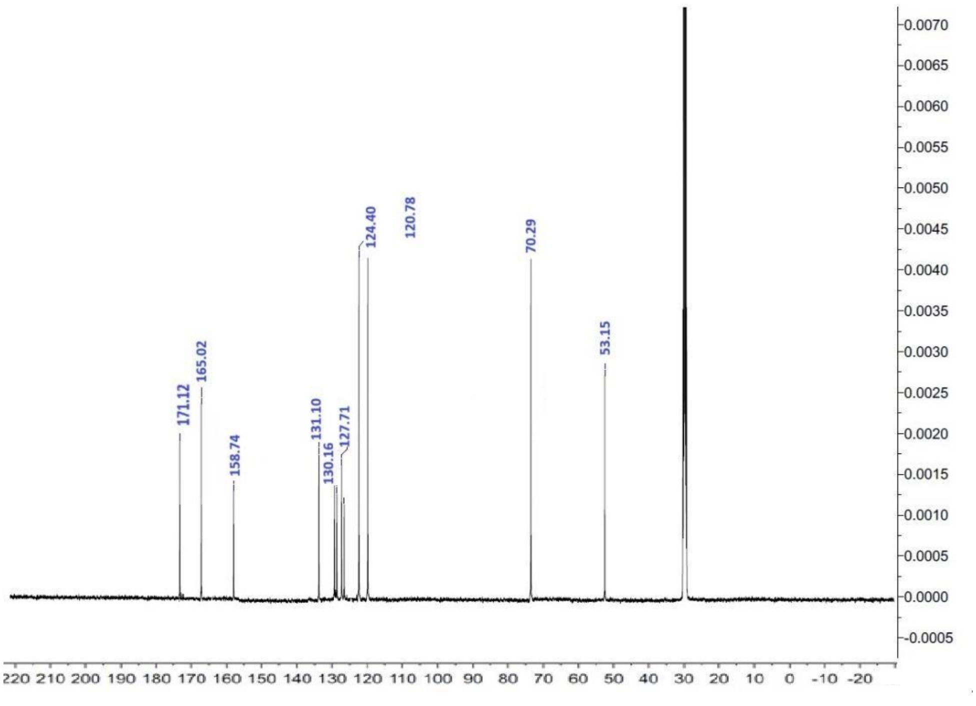

Figure S27. ^{13}C NMR spectrum of compound (9)

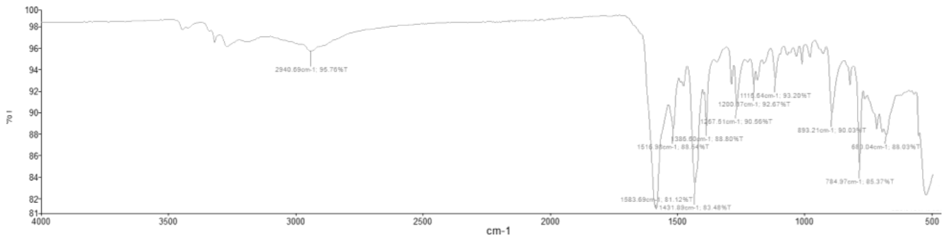

Figure S28. FTIR spectrum of compound (10)

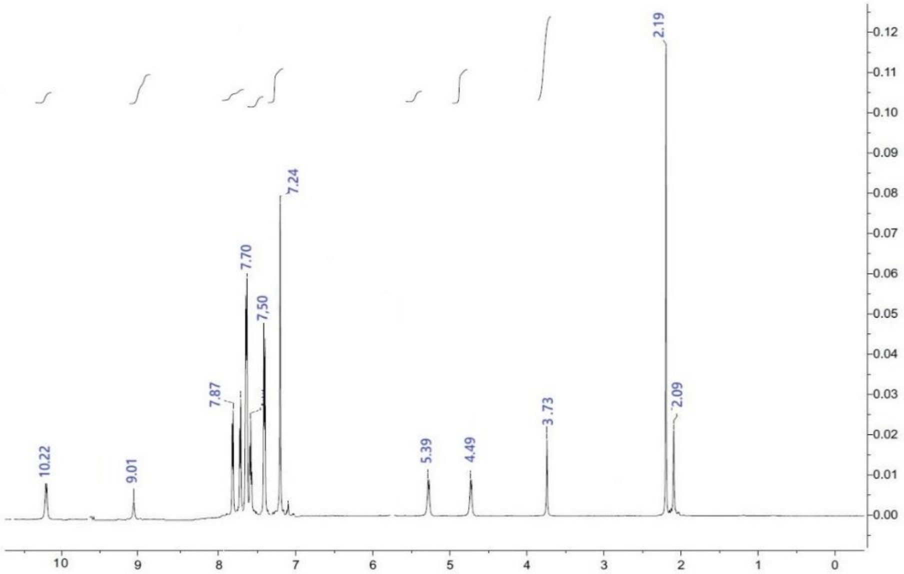

Figure S29. ^{1}H NMR spectrum of compound (10)

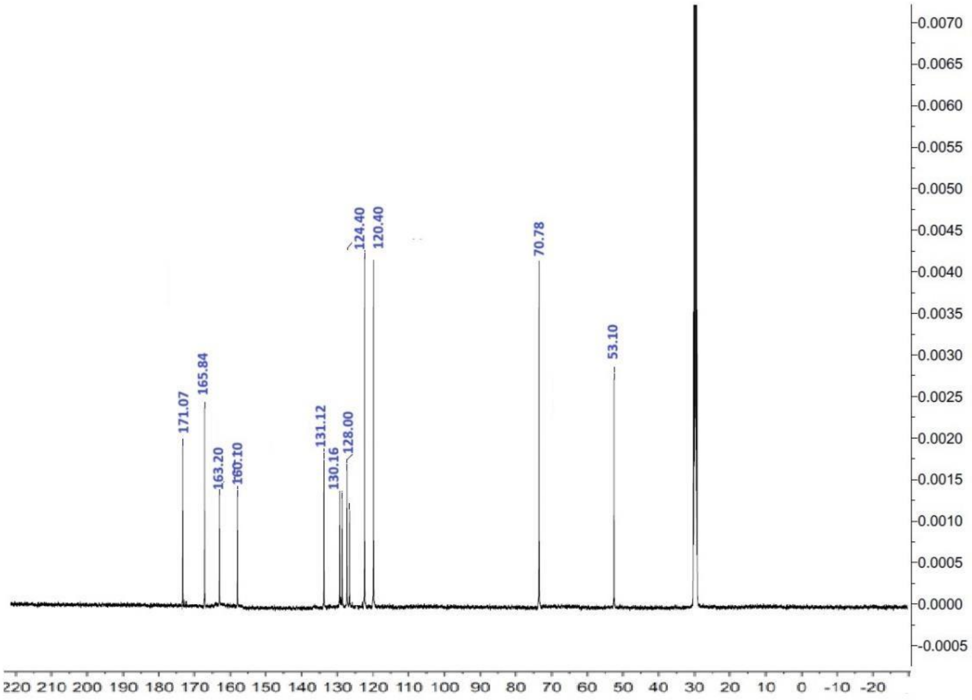

Figure S30. ^{13}C NMR spectrum of compound (10)

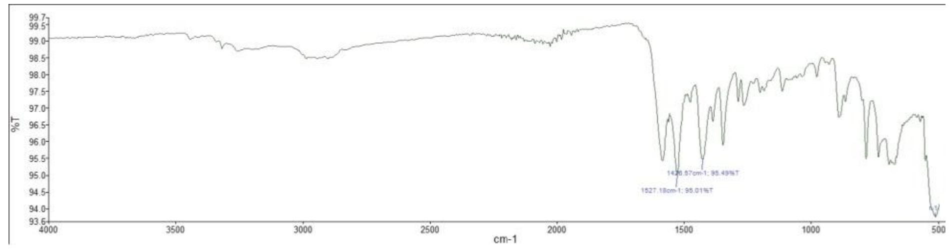

Figure S31. FTIR spectrum of compound (11)

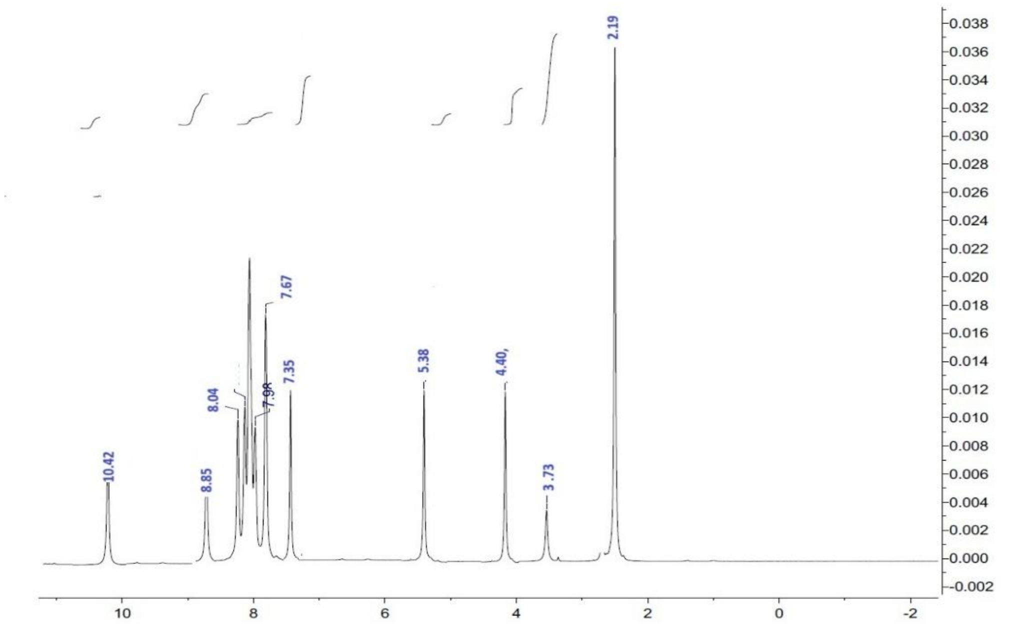

Figure S32. ^{1}H NMR spectrum of compound (11)

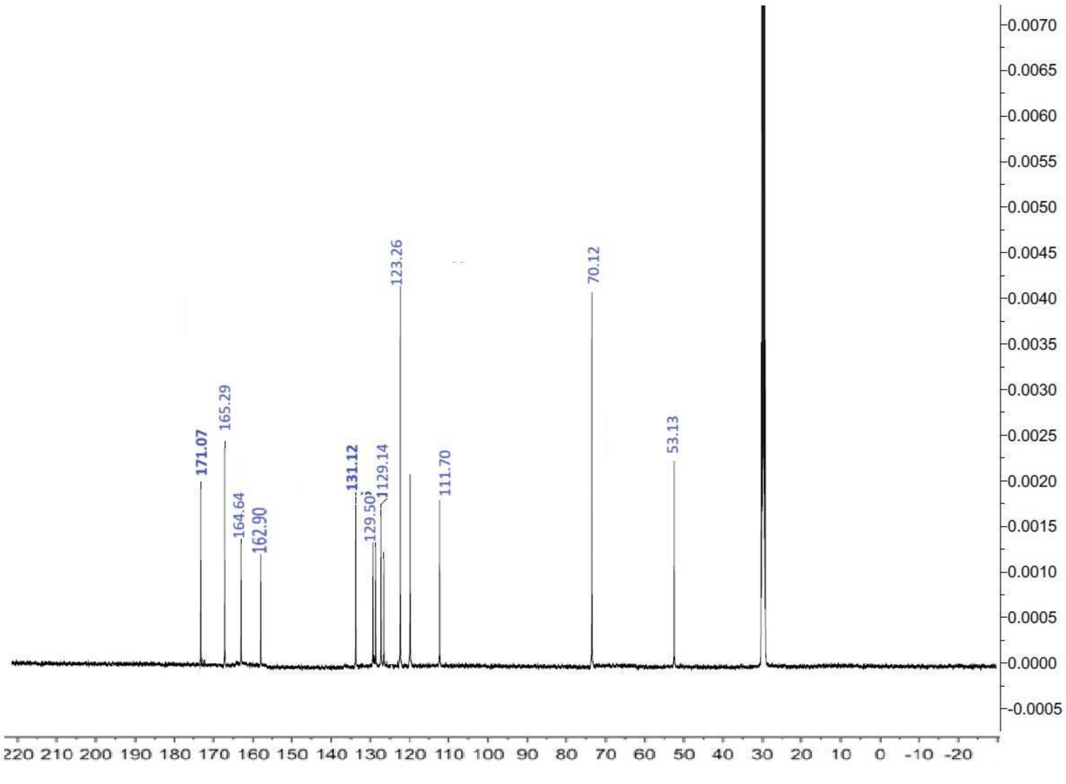

Figure S33. ^{13}C NMR spectrum of compound (11)

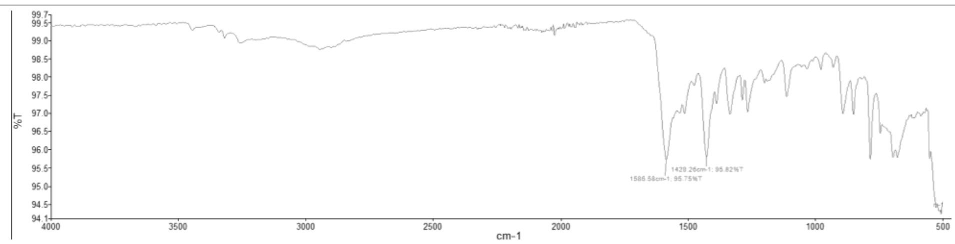

Figure S34. FTIR spectrum of compound (12)

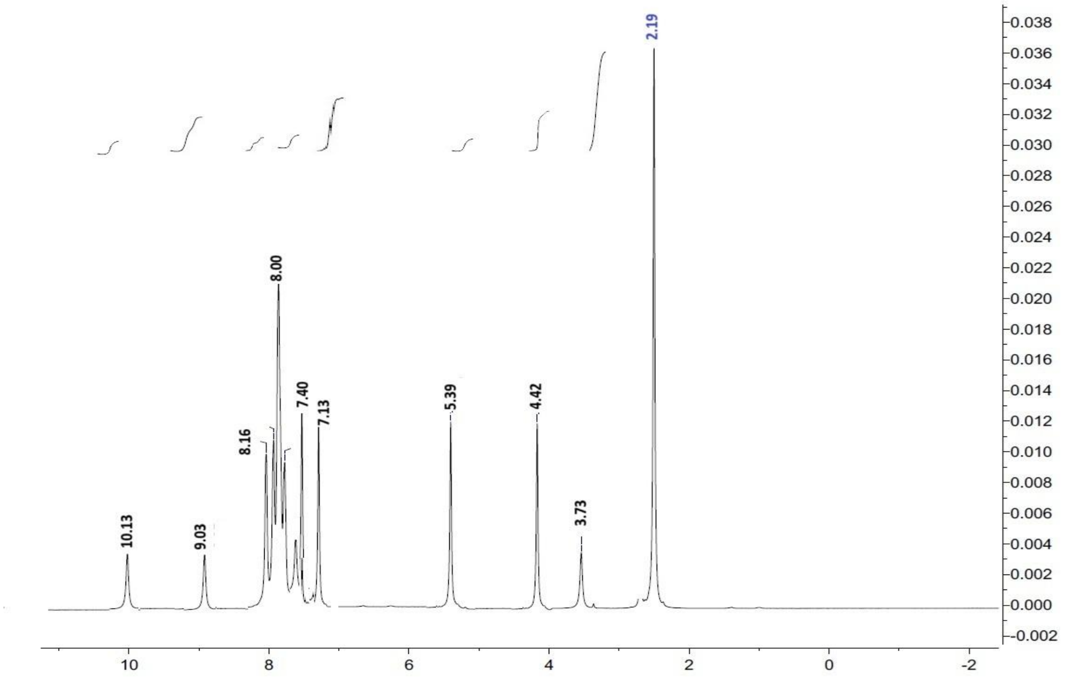

Figure S35. ^{1}H NMR spectrum of compound (12)

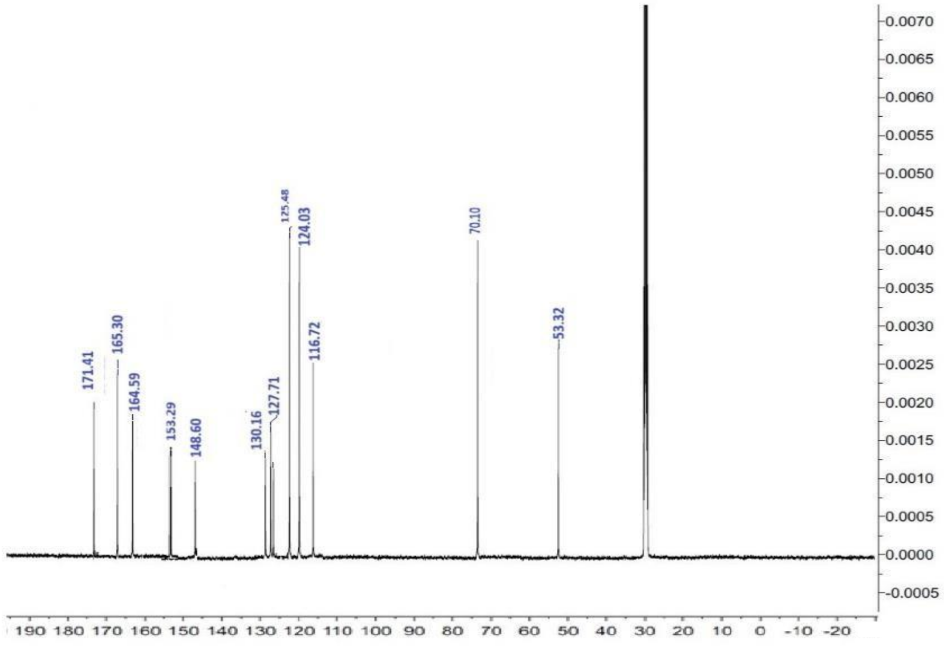

Figure S36. ^{13}C NMR spectrum of compound (12)
